# Single-cell and spatial analyses revealed the co-location of cancer stem cells and SPP1+ macrophage in hypoxic region that determines the poor prognosis in hepatocellular carcinoma

**DOI:** 10.1038/s41698-024-00564-3

**Published:** 2024-03-23

**Authors:** Guangyu Fan, Tongji Xie, Lin Li, Le Tang, Xiaohong Han, Yuankai Shi

**Affiliations:** 1 Department of Medical Oncology, National Cancer Center/National Clinical Research Center for Cancer/Cancer Hospital, Chinese Academy of Medical Sciences & Peking Union Medical College, Beijing Key Laboratory of Clinical Study on Anticancer Molecular Targeted Drugs; No. 17 Panjiayuan Nanli, Chaoyang District, Beijing, 100021 China; 2 Department of Pathology, National Cancer Center/National Clinical Research Center for Cancer/Cancer Hospital, Chinese Academy of Medical Sciences & Peking Union Medical College, Beijing, 100021 China; 3 Clinical Pharmacology Research Center, Peking Union Medical College Hospital, State Key Laboratory of Complex Severe and Rare Diseases, NMPA Key Laboratory for Clinical Research and Evaluation of Drug, Beijing Key Laboratory of Clinical PK & PD Investigation for Innovative Drugs, Chinese Academy of Medical Sciences & Peking Union Medical College; No.1, Shuaifuyuan, Dongcheng District, Beijing, 100730 China

**Keywords:** Cancer microenvironment, Tumour biomarkers, Cancer stem cells

## Abstract

In hepatocellular carcinoma (HCC), classical cancer stem cells (CSC) markers were shared by normal stem cells, targeting which may hinder hepatic regeneration and cause liver failure. Additionally, the spatial structure of CSC still remained elusive. To address these limitations, we undertook a comprehensive study combining single-cell data (56,022 cells from 20 samples) and spatial data (38,191 spots from eight samples) to obtain CSC signature and uncover its spatial structure. Utilizing the CytoTRACE algorithm, we discretely identified CSC, which displayed upregulated proliferation pathways regulated by HIF1A. A CSC signature of 107 genes was then developed using Weighted Gene Co-expression Network Analysis (WGCNA). Notably, HCC patients with high CSC levels exhibited an accumulation of SPP1+ macrophages (Macro_SPP1) expressing metalloproteinases (MMP9, MMP12, and MMP7) regulated by HIF1A, suggesting a hypoxic tumor region connecting Macro_SPP1 and CSC. Both CSC and Macro_SPP1 correlated with worse prognosis and undesirable immunotherapy response. Spatial analysis revealed the co-location of CSC and Macro_SPP1, with CD8 T cells excluded from the tumor region. The co-location area and non-tumor area of boundary exhibited a high level of hypoxia, with the HAVRC2 checkpoint highly expressed. Within the co-location area, the SPP1 signaling pathway was most active in cell-cell communication, with SPP1-CD44 and SPP1-ITGA/ITGB identified as the main ligand-receptor pairs. This study successfully constructed a CSC signature and demonstrated the co-location of CSC and Macro_SPP1 in a hypoxic region that exacerbates the tumor microenvironment in HCC.

## Introduction

Hepatocellular carcinoma (HCC) is the predominant subtype of primary liver cancer and ranks as the fourth leading cause of cancer-related mortality^1^. Immunotherapy has revolutionized cancer treatment, bringing significant therapeutic advancements for HCC patients^2,3^. Nevertheless, only a small proportion of patients respond to this treatment, underscoring the necessity to identify factors hindering immune infiltration and develop combination strategies to overcome immune resistance^4^.

Cancer stem cells (CSC) play a pivotal role in tumor initiation, progression, and metastasis^5,6^. Emerging evidence indicates that CSC can enhance mechanisms of immune evasion and promote the expansion of protumorigenic immune phenotypes, revealing the elimination of CSC clones of critical importance^7,8^. However, the current understanding of liver CSC has largely been based on the biology of normal stem cells^9^. Many of the identified CSC markers (CD13, CD24, EPCAM, CD44, and CD133) were shared by normal stem cells, and thus eradication of liver CSC through targeting these markers might result in the reduction of normal hepatic stem cells, which might inhibit hepatic regeneration and lead to liver failure^9–11^. Therefore, it is imperative to identify markers that are uniquely expressed in liver CSC.

Moreover, early research on liver CSC primarily relied on identifying markers through cell sorting and xenotransplantation in immunodeficient mice, resulting in limited resolution and overlooking heterogeneity^5^. Recent two studies have leveraged single-cell RNA sequencing (scRNA-Seq) to characterize CSC in HCC cell lines, tumors tissues, and xenografts^12,13^. The first study analyzed three established CSC markers (CD24, CD133, and EPCAM) and found that gene signatures linked to CD133 and EPCAM are independent predictors of HCC survival^13^. Another study identified one EPCAM+ population displaying a specific oncogenic gene expression signature^12^. Although valuable, these investigations primarily explored well-known CSC markers, highlighting the need for a new approach to derive a comprehensive CSC signature.

The tumor microenvironment (TME) plays a crucial role in regulating cancer stemness in HCC^14,15^. Liver CSC reside in dedicated niches where they interact reciprocally with cells and factors in the TME to regulate stemness. These interactions occur through diverse mechanisms, including cell-to-cell crosstalk, secreted factors, cell-matrix interactions, and unique biophysical properties of the niche, such as hypoxia, nutrient deprivation (especially glucose), and extracellular matrix (ECM) remodeling^14,16,17^. Furthermore, accumulating evidence suggests that specific immune cell types are instrumental in driving CSC expansion, mediating CSC-specific evasion of immune detection and destruction and promoting protumorigenic immune cell activities^18^. Unraveling the intricate web of CSC-TME interactions holds immense promise in deciphering the underlying mechanisms governing cancer stemness in HCC. To address this, spatial transcriptomics (ST) has emerged as a groundbreaking technology, enabling researchers to examine spatial information and gene expression profiles within tissues^19,20^. Leveraging ST, we can delve into the complexities of intratumor architecture and identify microenvironmental niches intricately associated with CSC. This empowers us to investigate specific locations enriched with CSC, explore the co-location of distinct TME cell subtypes alongside CSC, and dissect intricate interactions among various cellular components.

In this study, we developed and validated a CSC signature through de-novo large-scale analysis of scRNA-Seq datasets, encompassing 20 samples and 56,022 cells. We explored enriched pathways and transcription factors (TFs) associated with CSC, investigating its adverse role in prognosis and immunotherapy. Additionally, we observed the accumulation of SPP1+ macrophages in HCC samples with high CSC levels, revealing a hypoxic region stimulating the interaction between CSC and SPP1+ macrophages. Furthermore, we utilized ST data from eight HCC patients to explore the spatial structures of their co-location and the specific cell-cell communication between the two groups. Overall, our findings unveil a robust CSC signature and provide insights into the specific spatial structures of CSC that promote immune resistance.

## Results

### The landscape of CSC in HCC

**Fig. 1:**
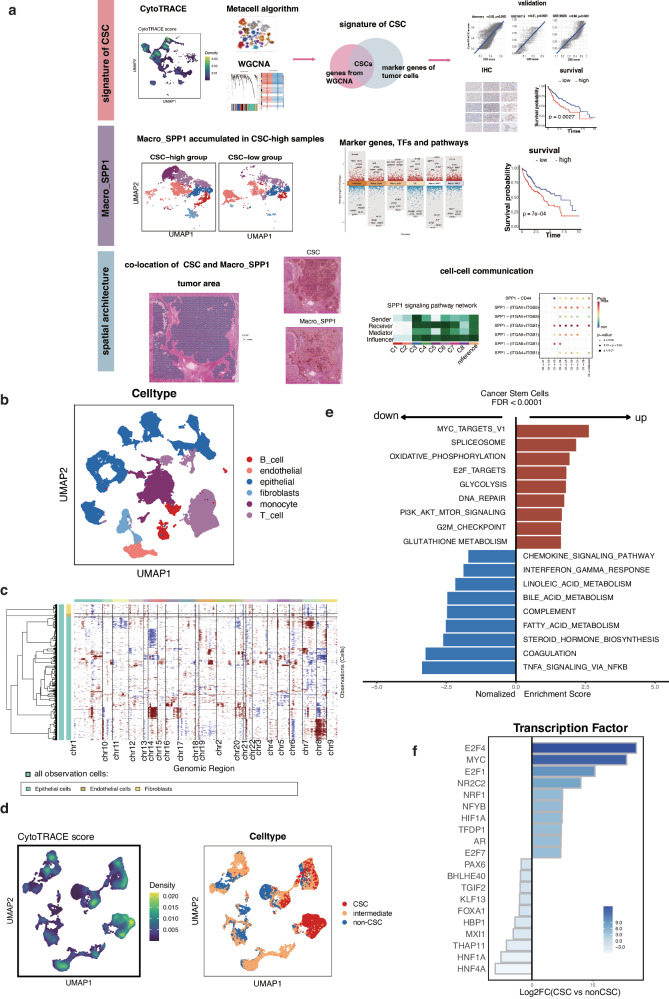
The landscape of cancer stem cells (CSC) in hepatocellular carcinoma (HCC) at the single-cell level. **a** Workflow depicting the study design. **b** Identification of six distinct clusters in HCC samples: epithelial cells, myeloid cells, fibroblasts, endothelial cells, T cells, and B cells. **c** Hierarchical heatmap illustrating large-scale copy number variations in cancer cells and spiked-in controls (fibroblasts and endothelial cells). The green bar to the far left represents all cells used as the observation cells, encompassing epithelial cells, spiked-in fibroblasts, and endothelial cells within the dataset.The different colors in the top row correspond to the 22 pairs of chromosomes on the x-axis. **d** UMAP plot displaying the distribution of CytoTRACE score in malignant cells and the three cell states (CSC, non-CSC and intermediate) (**e**) Bar chart showing the up-regulated and down-regulated pathways in CSC. **f** Expression patterns of the 20 most varied transcription factors (TFs) in CSC.

The workflow of this study is illustrated in Fig. 1a. First, the landscape of CSC in HCC was investigated. A total of 45 HCC samples from GSE151530 and GSE149614 were analyzed, resulting in the identification of 81,508 cells for single-cell analysis. The cells were categorized into six main clusters based on curated classical markers: epithelial cells, myeloid cells, fibroblasts, endothelial cells, T cells, and B cells (Fig. 1b). The inferCNV analysis was performed to discrete malignant cells. From the heatmap, no epithelial subclusters showed similar copy number variation (CNV) patterns as the spiked-in control cells, so all epithelial cells were labeled as malignant cells (Fig. 1c). 25 samples were excluded from further analysis due to a low number of malignant cells (*n* < 100), resulting a total of 56,022 cells from 20 samples for further analysis,.

To quantify the stemness level of malignant cells, the CytoTRACE score developed by Gulati et al. was computed, which was an robust computational framework for predicting differentiation states from scRNA-seq data, based on estimating the number of expressed genes per cell and constructing the differentiation hierarchies^21^. The CytoTRACE scores range from 0 to 1, with higher scores indicating higher stemness (lower differentiation) and vice versa. The CytoTRACE score presented great heterogeneity in tumor cells (Fig. 1d). Malignant cells with the highest and lowest 25% CytoTRACE score were classified as CSC and non-CSC, respectively. The presentation of CSC, non-CSC, and intermediate tumor cell populations on the UMAP plot was depicted in Fig. 1d. We calculated both the cell number and proportion of CSC in each sample from the scRNA-seq datasets, and the results are depicted in Figure S1A. Notably, the proportions of CSCs exhibited variations between samples. It is crucial to note that each sample corresponds to a specific portion of the tumor area rather than representing the entire patient. CSC showed distinct upregulation of proliferation-related pathways, such as the G2M checkpoint, MYC checkpoint, and E2F targets (Fig. 1e). Notable changes in CSC metabolic patterns were also observed, with downregulation of lipid metabolism-related pathways (fatty acid, bile acid, and steroid) and upregulation of glycolysis. Additionally, immune-related pathways (TNFA signaling, chemokine signaling, and complement) were found to be suppressed in CSC. Leveraging the slingshot algorithm from the R package slingshot, we inferred cell lineages and pseudotimes from single-cell gene expression data^22^. As anticipated, the pseudotime trajectory corroborated the characteristics of CSC, with CSC positioned at the beginning of the lineage and non-CSC located at the end of the trajectory (Figure S1b).

We also examined the activity of TFs in promoting uncontrolled self-renewal and tumor-initiating potential in CSC. Dorothea was used to explore potential variations in regulon activity between CSC and non-CSC, revealing distinct regulon activities^23^. The expression patterns of the 20 most varied TFs in cellular populations were illustrated (Fig. 1f). Upregulation of MYC family (MYC) and E2F family (E2F4, E2F1, and E2F7) was observed in CSC, which are key factors in cell proliferation, and differentiation^24^. HIF1A, a well-known hypoxia TF, exhibited enhanced regulon activities in CSC^25^. NRF1 also exhibited increased activities, which is an indispensable redox-determining factor for mitochondrial homeostasis^26^.

### The signature of CSC was identified using weighted gene co-expression network analysis

**Fig. 2:**
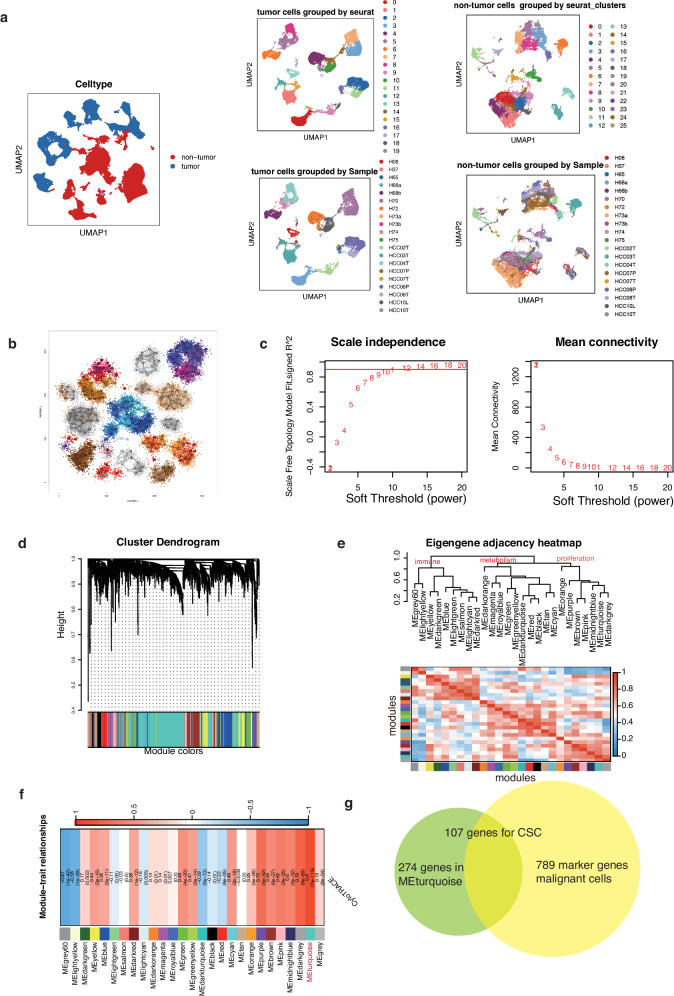
Identification of the cancer stem cells (CSC) signature using weighted gene co-expression network analysis (WGCNA). **a** UMAP plots showing the grouping of malignant cells (left) and non-tumor cells (right) based on sample origins, respectively. **b** Partitioning of 25,839 malignant cells into 320 metacells. Metacell clusters with the same color had relatively similar expression patterns. **c** Construction of a scale-free topology model with a soft thresholding power set to 9. **d** Hierarchical cluster tree revealing a total of 27 modules identified by WGCNA. **e** The upper dendrogram plot exhibits the hierarchical clustering of modules obtained from the clustering analysis depicted in Fig. 2d. The branches in the dendrogram represent the grouping of modules that display positive correlation. Meanwhile, the lower heatmap plot illustrates the adjacencies among all modules. Each row and column in the heatmap corresponds to a specific module, denoted by color. In the heatmap representation, the color scheme denotes the degree of adjacency between modules. The blue color signifies low adjacency, indicating a negative correlation, while red denotes high adjacency, indicating a positive correlation. **f** Heatmap displaying the correlation of each module with the CytoTRACE score, with the first number in each grid denoting the correlation coefficient and the second reflecting the *p*-value. **g** Identification of 107 CSC-related genes as the signature of CSC by intersecting the marker genes of malignant cells with the genes in the MEturquoise module.

We then aimed to identify the signature of CSC in HCC. We observed that malignant cells formed distinct clusters corresponding to their sample origin, while non-malignant cells showed no discernible variations across patients (Fig. 2a). Interestingly, malignant cells from the same patient often belonged to different clusters. These findings indicated the considerable intra-patient and inter-patient heterogeneity of malignant cells. To address the bias caused by this heterogeneity in cell selection for further weighted gene co-expression network analysis (WGCNA) analysis, we employed the non-parametric K-nearest neighbor (KNN) graph algorithm in the Metacell package to divide cells into homogeneous groups^27^. Following quality control, we retained 25,839 malignant cells and divided them into 320 metacells (Fig. 2b). Metacell clusters with the same color had relatively similar expression patterns. We summarized the relationship between the 320 metacells and the original sample in Figure S1c. These metacells, which grouped cells with similar transcriptional states, can serve as building blocks for approximating gene expression distributions in single-cell data.

Phenotype plasticity model suggests that CSCs are a dynamic subpopulation of cancer cells rather than a stable cell population. Accumulating reports also reveal that certain cancer cells can exhibit plasticity via a reversible transitioning between the CSC and non-CSC state, which repopulates the CSC pool and enables the cells to survive therapy. Using WGCNA, we investigated a nuanced examination of gene expression patterns along the continuum between CSC and non-CSC states. Initially, the expression profiles of the top 5000 variable genes from the 320 metacells were included. To construct a scale-free topology model, we set the soft thresholding power to 9 (Fig. 2c). After weight-based filtering, we obtained a total of 27 modules and their hierarchical cluster tree was depicted in Fig. 2D. The epigengene adjacent heatmap depicted the intermodule correlations among all modules (Fig. 2e). We then investigated the correlation between each module and CytoTRACE score, which represented the stemness level. Among these modules, MEturquoise (correlation = 0.9, *p* = 7e−115) showed the most significant association with the CytoTRACE score (Fig. 2f). The genes encompassed within the METurquoise module were found to be associated with numerous proliferation-related pathways. These pathways include the G2M checkpoint, MYC checkpoint, and E2F targets, all of which are consistently associated with the features of CSCs. We intended to construct the tumor specific stemness features, so we recognized differentially expressed genes (DEGs) of tumor cells compared to other types of stroma and immune cells (avg_logFC > 0.5, *P* < 0.05). By intersecting the marker genes of malignant cells with the genes in the MEturquoise module, we identified 107 CSC-related genes, which constituted the CSC signature (Fig. 2g, Table S1).

### CSC correlated with worse prognosis and undesirable immunotherapy response

**Fig. 3:**
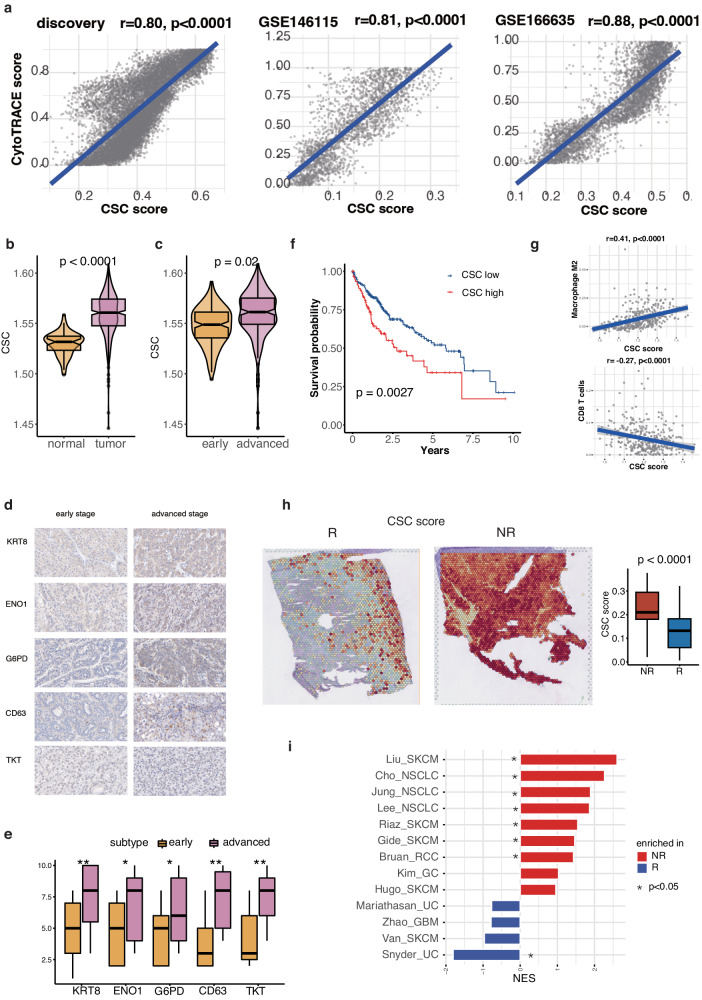
Correlation of cancer stem cells (CSC) with worse prognosis and undesirable immunotherapy response. **a** Scatter plots showing the correlation between the identified CSC signature and CytoTRACE score in the discovery dataset and two validation datasets (GSE166635, GSE146115). **b** Distribution of CSC in normal and HCC tissues. **c** Distribution of CSC in early and advanced stage HCC tissues. **d** Immunohistochemistry images of five proteins identified in the CSC signature (ENO1, TKT, CD63, G6PD, and KRT8) in our cohort consisting of 32 HCC patients. (**e**) Boxplots displaying the distribution of the five proteins in early and advanced stage samples. Statistical significance (*p* value): ‘*’ for ‘between 0.05 and 0.01’, ‘**’ for ‘between 0.01 and 0.001’ and ‘***’ for ‘lower than 0.001’. **f** HCC patients in the TCGA cohort with higher CSC infiltration exhibited shorter overall survival (OS). **g** CSC score showed positive correlation with Macrophage M2 and negative correlation with CD8 T cells. **h** The non-responders exhibited higher CSC scores compared to responders in HCC patients treated with immunotherapy. **i** Pathway enrichment analysis revealing that the signature of CSC was enriched in non-responders in multiple immunotherapy cohorts.

Subsequently, to test the reliability of our our identified CSC signature, we calculated it as the CSC score for each malignant cell using the AUCell algorithm^28^. In our discovery dataset (combined of GSE151530 and GSE149614), the correlation between the CSC score and CytoTRACE score reached 0.80, with a p-value < 0.0001 (Fig. 3a). To validate this correlation, we recruited two additional single-cell datasets of HCC (GSE166635, GSE146115), and the correlation between the two scores across two datasets was 0.88 and 0.81, respectively (Fig. 3a). These investigations provided evidence for the validity of the 107 WGCNA-identified genes and their functional relevance. In addition, we incorporated two stemness-related signatures from prominent databases (KEGG and REACTOME), which could be found in Table S2. These gene sets were then utilized to assess and cross-validate the identified CSC signature across three datasets. We calculated the scores for these curated signatures and observed a significantly positive correlation between each signature and our identified CSC score across all analyzed datasets (Figure S1d).

To explore the role of CSC in bulk level, we analyzed data from the The Cancer Genome Atlas (TCGA) cohort and observed significantly upregulated infiltration of CSC in tumor samples compared to adjacent normal tissues (Fig. 3b). Furthermore, the infiltration of CSC increased with advancing stages of HCC (Fig. 3c). We also performed immunohistochemistry (IHC) of five proteins (ENO1, TKT, CD63, G6PD and KRT8) highly expressed by CSC on our in-house cohort of 32 HCC patients. We have provided a comprehensive summary of the clinical information for single-cell discovery cohort and our in-house cohorts in Table S3. The clinical information incorporated age, sex, viral hepatitis status, and pathological stage (AJCC staging system) for both cohorts. Following statistical analysis using Pearson’s Chi-squared test, no significant differences were observed in the clinical characteristics between the two cohorts. Consistent with these results, IHC staining confirmed their association with advanced stages in this cohort (Fig. 3d and e). Moreover, HCC patients in the TCGA cohort with higher infiltration of CSC exhibited shorter overall survival (OS) (Fig. 3f).

Furthermore, we observed significant TME component changes related to CSC remodeling. Macrophage M2, a subtype of immunosuppressive myeloid cells had positive correlation with CSC (*r* = 0.41, *p* < 0.0001), while CD8+ T cells negatively associated with CSC (*r* = −0.27, *p* < 0.0001), indicating the potential interaction between CSC and Macrophage M2 to impede anti-tumor cells infiltration (Fig. 3g). We incorporated ST data from a recent study GSE238264 focused on HCC patients treated with immunotherapy. The dataset included samples from a clinical trial of neoadjuvant cabozantinib (a multi-tyrosine kinase inhibitor that primarily blocks VEGF) and nivolumab (a PD-1 inhibitor), with responders and non-responders distinguished based on treatment outcomes. Notably, the non-responders exhibited higher CSC scores compared to responders, strengthening our analysis and underscoring the potential relevance of CSC in immunotherapeutic responses (Fig. 3h). As for immunotherapy, gene set enrichment analysis (GSEA) revealed that CSC was enriched in non-responders across seven immunotherapy cohorts, including non-small cell lung cancer (NSCLC), skin cutaneous melanoma (SKCM), renal cell carcinoma (RCC) (Fig. 3i). The detailed information of immunotherapy cohorts used in our study could be obtained from Table S4. This result provided additional evidence supporting the adverse role of CSC in the context of immunotherapy.

### Macro_SPP1 accumulated in CSC-high group

**Fig. 4:**
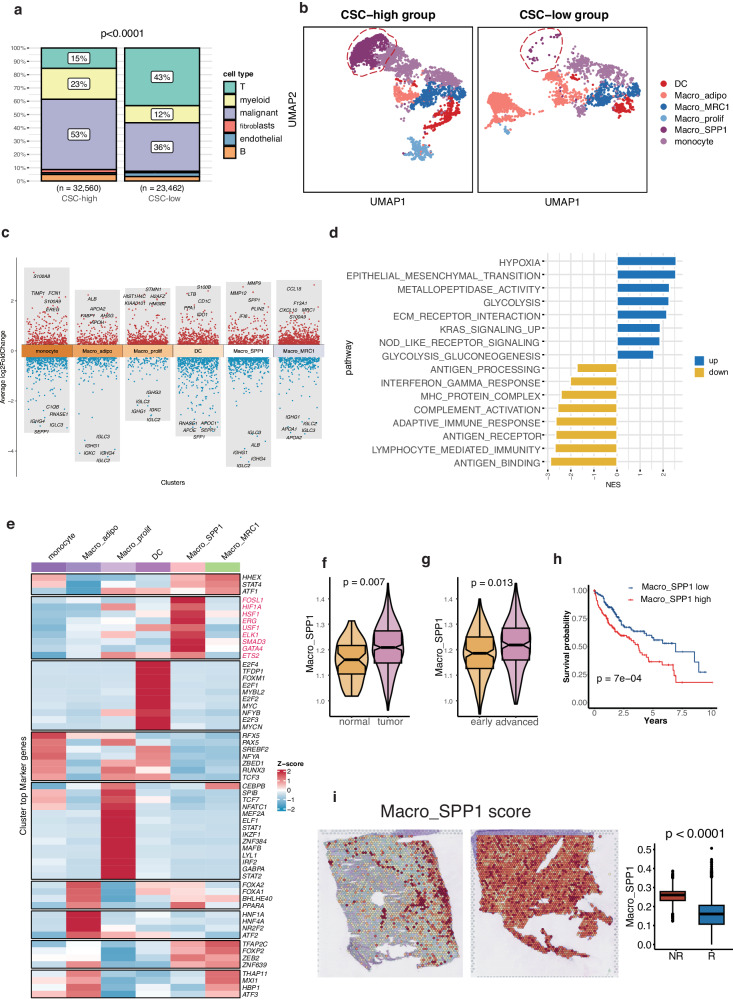
SPP1+ macrophage (Macro_SPP1) expressed metalloproteinases, and regulated by HIF1A. **a** The proportion of six clusters (epithelial cells, myeloid cells, fibroblasts, endothelial cells, T cells, and B cells) in the CSC-high and CSC-low groups. **b** UMAP plots showing myeloid cell subtypes in the CSC-high and CSC-low groups, including DC, monocyte, Macro_SPP1, MRC1+ macrophage (Macro_MRC1), adipogenic macrophage (Macro_adipo), and proliferating macrophage (Macro_prolif). The red circle encompassed Macro_SPP1. **c** Top 5 upregulated and downregulated genes of six subclusters. **d** Bar chart displaying the up-regulated and down-regulated pathways in Macro_SPP1. **e** Expression patterns of the most varied transcription factors (TFs) in Macro_SPP1. (**f**) Distribution of Macro_SPP1 in normal and HCC tissues. **g** Distribution of Macro_SPP1 in early and advanced stage HCC tissues. **h** HCC patients with higher infiltration of Macro_SPP1 exhibited shorter overall survival (OS). (**i**) The non-responders exhibited higher Macro_SPP1 compared to responders in HCC patients treated with immunotherapy.

We proceeded to investigate the impact of CSC on the TME. The CSC score for each sample was defined as the mean CSC score of all malignant cells within the sample. We have included a visualization depicting the CSC scores per sample in Figure S2a. This plot provides a comprehensive representation of CSC scores across various samples, enabling an overview of the distribution and variation in cancer stemness among different samples. Based on the median CSC score of all samples, we divided the 20 samples into CSC-high and CSC-low groups. Notably, these two groups exhibited distinct landscapes of TME components (Fig. 4a). The proportion of malignant cells was significantly higher in the CSC-high group (*n* = 0.53) compared to the CSC-low group (*n* = 0.36). Importantly, we observed a higher infiltration of myeloid cells in the CSC-high group (*n* = 0.23) compared to the CSC-low group (*n* = 0.12), while T cells exhibited greater infiltration in the CSC-low group (*n* = 0.43) compared to the CSC-high group (*n* = 0.15). However, there were no significant differences in the proportions of B cells, endothelial cells, and fibroblasts between the two groups.

The enhanced infiltration of myeloid cells in HCC patients suggests their functional roles in promoting CSC initiation. We subsequently focused on the alterations in the subtypes of myeloid cells between the two groups. Initially, we divided the myeloid cells (*n* = 10,603) into six subclusters, including DC, monocyte, SPP1+ macrophage (Macro_SPP1), MRC1+macrophage (Macro_MRC1), adipogenic macrophage (Macro_adipo), and proliferating macrophage (Macro_prolif). The result revealed a predominant presentation of Macro_SPP1 populations in CSC-high tissues (Fig. 4b). Macro_SPP1 was reported to be one subtype of Macrophage M2^29^. Macro_SPP1 represents CSC-specific macrophages and accounted for 23% of myeloid cells in CSC-high samples, while only constituting 1.8% of myeloid cells in CSC-low tissues. On the other hand, Macro_adipo, an adipogenic myeloid cell subtype expressing lipid metabolism-related genes (ALB, APOA2, FABP1, and APOH), showed greater infiltration in the CSC-low group, accounting for 52.5% of myeloid cells in CSC-low samples compared to 18.8% in CSC-high tissues. We calculated the cell proportion of both CSC and SPP1+ macrophages in each sample from the scRNA-seq datasets. Our analysis revealed a positive correlation in cell proportion between these two populations, further supporting the association observed in the spatial transcriptomics data (Figure S2b).

### Macro_SPP1 expressed metalloproteinases and regulated by HIF1A

Macro_SPP1 exhibits distinct functions compared to other myeloid subtypes. The marker genes of Macro_SPP1 could be found in Table S5. Notably, Macro_SPP1 demonstrates higher expression levels of metalloproteinases such as MMP9, MMP12, and MMP7, which are involved in extracellular matrix remodeling and immune response regulation^30^ (Fig. 4c). In addition, Macro_SPP1 expresses chemokines like CXCL3, CCL20, CCL7 and CXCL8. The DEGs highly expressed in Macro_SPP1 are enriched in hypoxia, ECM-receptor interactions, epithelial-mesenchymal transition (EMT), metallopeptidase activity, KRAS signaling up, and glycolysis (Fig. 4d). However, immune-related pathways, including antigen processing and presentation, lymphocyte-mediated immunity, and complement activation, are significantly suppressed in Macro_SPP1. These findings suggest that Macro_SPP1 may play a role in promoting EMT and suppressing immune responses in HCC.

Furthermore, the activity of Macro_SPP1 is potentially regulated by the transcription factor HIF1A, a key factor in the hypoxia-induced pathway^25^ (Fig. 4e). Another transcription factor, FOSL1, is also expressed by Macro_SPP1 and is known to regulate invasion and metastasis, linking with EMT and cancer cell stemness^31^. HSF1 was able to protect cells from stress, averting proteomic instability and repressing tumor-suppressive amyloidogenesis^32,33^. These findings suggest the activation of these transcription factors involved in the commitment of Macro_SPP1. Given that both CSC and Macro_SPP1 are implicated in the hypoxia-induced pathway, we hypothesize the presence of a localized network within the hypoxic region of the tumor that connects Macro_SPP1 and CSC, collaborating to exacerbate the HCC microenvironment.

To validate the significance of Macro_SPP1 infiltration, we analyzed data from the TCGA cohort and observed significantly upregulated infiltration of Macro_SPP1 in tumor samples compared to adjacent normal tissues (Fig. 4f). The infiltration of Macro_SPP1 increased with advancing stages of HCC (Fig. 4g). Moreover, HCC patients in the TCGA cohort with higher infiltration of Macro_SPP1 exhibited shorter OS (Fig. 4h). Notably, the non-responders in the immunotherapy cogort exhibited higher Macro_SPP1 compared to responders, strengthening our analysis and underscoring the potential relevance of Macro_SPP1 in immunotherapeutic responses (Fig. 4i). As for immunotherapy, gene set enrichment analysis (GSEA) revealed that Macro_SPP1 was enriched in non-responders across six immunotherapy cohorts, including NSCLC, urothelial carcinoma (UC), RCC and gastric cancer (GC) (Figure S2c).

### Co-location of CSC and Macro_SPP1 revealed by spatial transcriptomics

To investigate the spatial organization of CSC and Macro_SPP1, we conducted spatial transcriptomics sequencing using tumor tissue sections from eight HCC patients. We obtained transcriptomics data with a median of 4900 spots for all samples. The median genes per spot across all samples was around 6000, and the percentage of mitochondrial genes across all samples were below 5% (Figure S3). The detailed quality information of eight ST samples could be found in Table S6.

Similar to the complexity encountered in single-cell data analysis, identifying malignant cells solely based on gene expression patterns in ST data proves challenging, especially in discerning between malignant and normal epithelial cells. To address this complexity, we performed the inferCNV analysis to specifically delineate malignant cells from other cell types based on their CNV patterns.

**Fig. 5:**
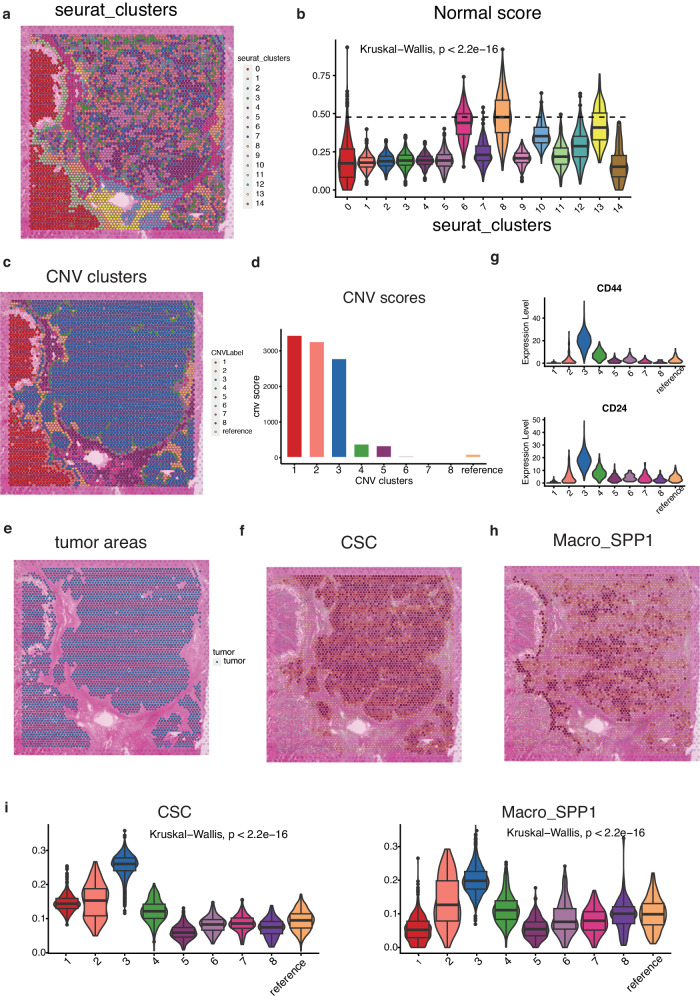
The co-location of cancer stem cells (CSC) and SPP1+ macrophage (Macro_SPP1) in tumor areas. **a** Clustering of 4825 spots in sample 1 into 14 distinct clusters. **b** Distribution of normal score in the 14 clusters. **c** Hierarchical clustering assigning all spots, except the reference cluster, into eight clusters. **d** Bar charts showing the distribution of copy number variation (CNV) score in the eight tumor clusters and reference clusters. **e** Identification of tumor areas. **f** Plot showing the distribution of CSC in tumor areas. **g** Expression of classic CSC marker CD24 and CD44 in the eight tumor clusters and reference clusters. **h** Plot showing the distribution of Macro_SPP1 in tumor areas. (**i**) Violin plot showing the distribution of CSC (left) and Macro_SPP1 (right) in the eight tumor clusters and reference clusters.

This procedure of inferCNV analysis included two clustering phase. Firstly, in the first clustering phase, we utilized the Seurat package to perform the pipeline of dimensionality reduction and clustering on ST data. After running PCA dimensional reduction, KNN algorithm in the FindClusters function of Seurat package was applied to cluster spatial spots, with .resolution = 0.8.We then took the sample 1 for example. 4825 spots in sample 1 were divided into 14 distinct clusters (Fig. 5a). The “Normal score” was calculated for each spot based on a series of immune-related signatures encompassing pan-immune markers (PTPRC), pan-T cell markers (CD2, CD3D, CD3E, CD3G), B cell markers (CD79A, MS4A1, CD79B), and myeloid cell markers (CD68, CD14), representing an average value for immune features within each spot. Cluster 8 in the first clustering analysis, which had the highest normal score, was designated as the reference cluster for inferCNV analysis (Fig. 5b).

Then, in the second clustering phase, hierarchical clustering based on tree partitioning then assigned all spots excepting the reference cluster into eight clusters based on CNV patterns (Fig. 5c). Clusters 1, 2, and 3 with high CNV score (all above 2000), were identified as malignant clusters, while the remaining clusters exhibited significantly lower CNV scores, all below 500 (Fig. 5d). We verified these annotations by consulting two independent pathologists who analyzed the HE histology information. Consistent with the CNV analysis, clusters 1, 2, and 3 corresponded to scattered tumor areas, whereas the other clusters primarily consisted of normal hepatocytes, fibroblasts, and mixed immune cells (Fig. 5e). This exploration confirmed the accurate identification of tumor areas.

To assess the presence of CSC and Macro_SPP1, we scored the spots in this sample using the signatures derived from the single-cell analysis. We observed substantial CSC heterogeneity within the sample, which was heavily influenced by their spatial patterns. Importantly, the CSC signature scores were mainly detected in tumor clusters (Fig. 5f). The three tumor clusters exhibited distinct CSC features, with cluster 3 having the highest CSC score (Fig. 5f). CD24 and CD44, the well-known CSC markers were highly expressed in cluster 3 (Fig. 5g). To distinguish between CSC and non-CSC cells within cluster 3, we utilized the cell type deconvolution method CARD from the R package CARD^34^. Given the variability in cell numbers within a single spot on the 10X Genomics Visium platform (ranging from 1 to 10 cells), it became evident that each spot could comprise a mixture of CSC, non-CSC, and the intermediate cell state. We observed the each spot contained varying proportion of three cell types. CSC cells were scattered throughout the tumor area rather than forming distinct clusters and mainly co-located with non-CSC and the intermediate cell state (Figure S4a). This pattern of scatter, coupled with the co-location feature of CSCs, likely contributed to the observed relatively close and relatively high CSC scores in many spots within cluster 3. The density plot of CSC score in all spots indicates the majority of spots in cluster 3 had higher CSC scores than other clusters and had relatively close CSC scores (Figure S4b).

We observed that Macro_SPP1 were closely situated near CSC in cluster 3 (Fig. 5h). Additionally, Macro_SPP1 also accumulated near the tumor boundary of cluster 3, constituting the immunosuppressive front for CSC maintenance. Cluster 3 displayed the highest level of CSC and Macro_SPP1 (Fig. 5i). This pattern of co-localization between Macro_SPP1 and CSC was consistently observed in the other seven samples as well (Figure S4c), suggesting a physical interaction between CSC and Macro_SPP1 within the niche.

### HIF1A and HAVCR2 highly expressed in the boundary of co-location area

**Fig. 6:**
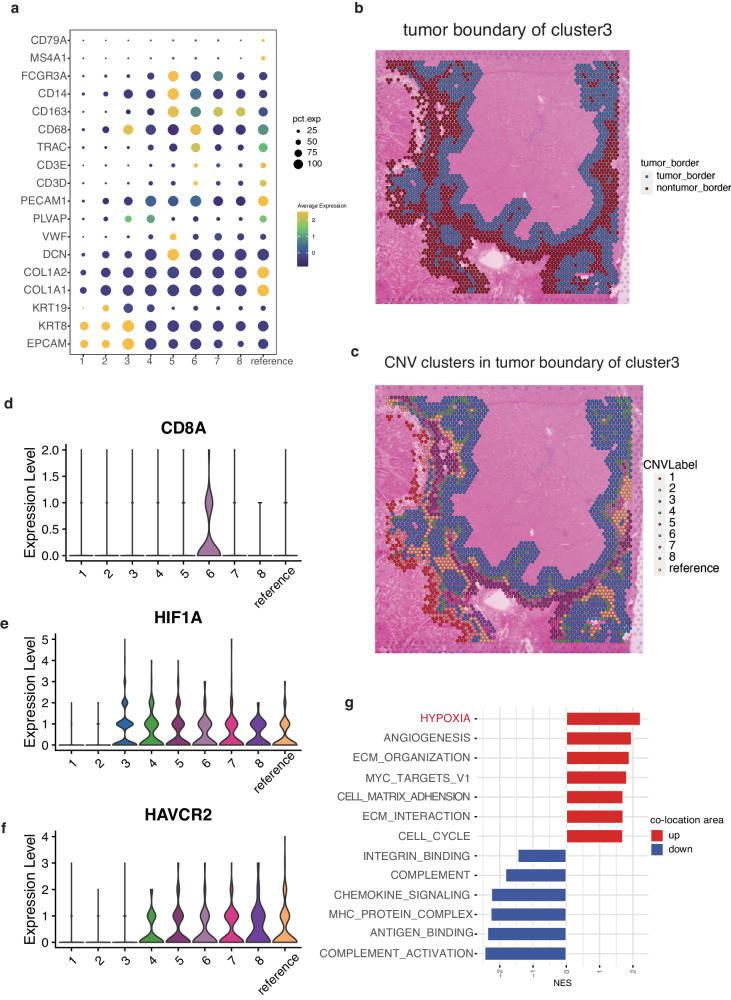
HIF1A and HAVCR2 highly expressed in the boundary of co-location area. **a** The marker genes of six cell types (epithelial cells, myeloid cells, fibroblasts, endothelial cells, T cells, and B cells) in the CNV clusters. **b** The nearest 3-spot width area near the outermost circle of cluster 3 was defined as its tumor boundary area, including the tumor and non-tumor area. **c** The CNV clusters in the tumor boundary of cluster 3. **d** The distribution of CD8A in CNV clusters in the tumor boundary of cluster 3. **e** The distribution of HIF1A in CNV clusters in the tumor boundary of cluster 3. **f** The distribution of HAVCR2 in CNV clusters in the tumor boundary of cluster 3. **g** The enriched pathways in the tumor and non-tumor area of cluster 3’s tumor boundary.

We then explored the phenotype of CNV clusters in sample 1. The marker genes of six cell types (epithelial cells, myeloid cells, fibroblasts, endothelial cells, T cells, and B cells) in the CNV clusters were displayed in Fig. 6a. EPCAM, KRT8 and KRT19 as marker genes of epithelial cells were highly expressed in clusters 1, 2, 3. Cluster 3 also showed high expression of CD68 (myeloid cells marker) and PLVAP (endothelial cell marker). Cluster 4 exhibited elevated expression of PLVAP. Cluster 5 showed infiltration by endothelial cells marked by VWF, myeloid cells marked by CD14, CD163 and FCGR3A, and fibroblasts marked by DCN. Cluster 6 was characterized by T cell accumulation (CD3D, CD3E, and TRAC) and myeloid cells (CD68), while clusters 7 and 8 were dominantly infiltrated by myeloid cells marked by CD163. The reference cluster displaying the highest normal score, contained immune and stromal cells such as fibroblasts (COL1A1 and COL1A2), endothelial cells (PLVAP and PECAM1), T cells (CD3D, CD3E, and TRAC), and B cells (MS4A1 and CD79A).

The tumor boundary is a niche composed of malignant cells in the outermost circle of solid tumor and non-malignant cells that are closely adjacent in spatial architecture, bridging these distinct spatial regions. To further elucidate the spatial architecture of CSC, we then investigated the boundary area of cluster 3. The nearest 3-spot width area near the outermost circle of cluster 3 was defined as its tumor boundary area, encompassing all clusters (Figs. 6b, c). CD8A, a marker of CD8 T cells, was only expressed in cluster 6, indicating the exclusion of anti-tumor immune cells from the tumor region (Fig. 6d). HIF1A, a well-known hypoxia factor, was observed in cluster 3 and its boundary clusters, highlighting the crucial role of hypoxia in remodeling the TME of CSC^25^ (Fig. 6e). Notably, PDCD1, CTLA4, and CD274, the important immunotherapy targets, showed almost no expression in all areas, while HAVCR2 exhibited significant expression in the boundary area of cluster 3, suggesting a potential immunotherapy target^35^ (Fig. 6f).

Furthermore, we identified enriched pathways in tumor and non-tumor area of the tumor boundary, which contributed to the niche. Pathways enriched in tumor area were mainly associated with ECM organization, angiogenesis, cell cycle and cell matrix adhesion (Fig. 6g). Furthermore, we observed that hypoxia was the most enriched pathway within the co-location area of CSC and SPP1+macrophages (Fig. 6g), signifying the significant interplay between these elements within the hypoxic microenvironment. This finding suggests that the co-localization of Macro_SPP1 and CSC may play a role in cell migration, adhesion, and ECM organization. Pathways enriched in non-tumor area were mainly associated with T cell-mediated cytotoxicity and lymphocyte-mediated immunity, chemokine signaling, and complement. Consequently, the desmoplastic microenvironment could potentially be regulated by the interaction between CSC and Macro_SPP1, leading to the limitation of immune cell infiltration into the tumor core.

### SPP1 signaling in cell-cell communication

**Fig. 7:**
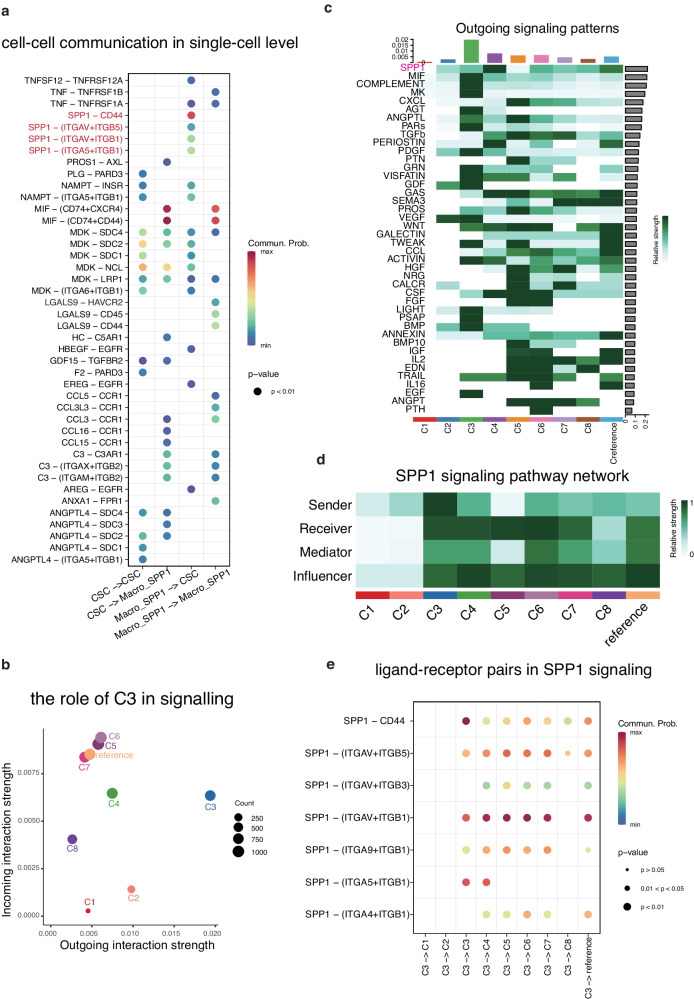
SPP1 signaling in cell-cell communication. **a** The cell-cell communication between CSC and Macro_SPP1 at the single-cell level. **b** The role of cluster 3 in the cell-cell communication in the tumor boundary area of cluster 3. (**c**) The outgoing pattern of the CNV clusters in the tumor boundary area of cluster 3. **d** The SPP1 signaling pathway network in the tumor boundary area of cluster 3. **e** The ligand-receptor pairs in SPP1 signaling pathway signaled by cluster 3 to all clusters.

We further proceeded to investigate the interactions between Macro_SPP1 and CSC and the impact of their co-location on the boundary area. CellChat was utilized to analyze communication interactions and identify communicating molecules at the single-cell level between CSC and other principal cell types. Since the 10X Genomics Visium platform could accommodate 1–10 cells in one spot, it was challenging to precisely recognize the interactions between Macro_SPP1 and CSC. Therefore, we first evaluated their putative crosstalk at the single-cell level (Fig. 7a). The SPP1 signaling pathway was observed in the communication between CSC and Macro_SPP1, where SPP1 interacted with CD44, ITGAV, ITGA5, ITGB1, and ITGB5 on CSC. CD44 was a well-known marker of CSC, revealing its new role in regulating TME remodeling. LGALS9-HAVCR2 was observed in the self-communication of Macro_SPP1, which was reported to illicit immunosuppression in TME^35^. Furthermore, MIF exhibited significantly high activity between the two groups, being signaled by CSC to activate CD74, CD44, and CXCR4 in Macro_SPP1. Moreover, chemokines (CCL3, CCL16, and CCL15) released from CSC targeted CCR1 expressed in Macro_SPP1. Taken together, these targets play important roles in immunosuppressive reactions.

Moving forward, our focus shifted to communication in the boundary area, with cluster 3 standing out as the most active cluster, engaged in numerous incoming and outgoing interactions (Fig. 7b). Thus, we centered our attention on intercellular communication between cluster 3 and all clusters. Cluster 3 activated various signaling pathways, including SPP1, MIF, COMPLEMENT, MK, and AGT (Fig. 7c). The SPP1 signaling pathway exhibited the highest relative strength in all communication activities. Cluster 3 emerged as the primary sender of the SPP1 signaling pathway, while cluster 3, 4, 5, 6, 7, and the reference cluster within the boundary area served as receivers and influencers of the SPP1 signaling (Fig. 7d). These findings consistently supported our single-cell level results, demonstrating SPP1’s interaction with CD44, ITGAV, ITGA5, ITGB1, and ITGB5 on CSC (Fig. 7e). Overall, these results underscore the vital role of the SPP1 signaling in mediating interactions between Macro_SPP1 and CSC and the consequential impact of their co-location on the boundary area. Additionally, we also observed MIF signaling pathway and chemokine-related interactions in communication between cluster 3 and all clusters (Figure S5). Notably, TGFB signaling demonstrated activities in the self-communication of cluster 3, suggesting immunosuppressive communication within the co-location area (Figure S5).

## Discussion

CSC have been implicated in fostering tumor progression, metastasis, and therapy resistance, leading to an immunosuppressive TME^5^. However, the investigation of CSC and their spatial organization in HCC remains limited. In this study, we developed a reliable CSC signature using single-cell data and intriguingly, observed the accumulation of Macro_SPP1 in proximity to CSC in ST data. Both CSC and Macro_SPP1 were linked to worse survival, underscoring their potential as prognostic biomarkers. Our findings provide insights into the modulation of the TME by CSC, guiding future trials and biomarker discovery.

At the single-cell level, we characterized the CSC signature and elucidated their phenotype. CSC exhibited significant proliferation, with upregulated proliferation-related pathways. Metabolically, CSC demonstrated anaerobic glycolysis and enhanced glutathione metabolism, a well-known antioxidant response^36,37^. Reactive oxygen species resulting from glutathione depletion hindered CSC self-renewal, promoting differentiation and rendering differentiated cancer cells susceptible to apoptosis^38,39^. Hypoxia could induce an increase in the expression of transcription factors (e.g., OCT4, SOX2, c-myc, and Nanog) contributing to CSC sustenance^40,41^. Hypoxia also has been linked to cisplatin resistance through an AKT-HIF1α-PDGF-BB autocrine signaling loop and enhanced HCC stemness and CD24 + HCC cell maintenance via both HIF1α-dependent and HIF2α-dependent mechanisms^42,43^. Furthermore, DNA repair played a pivotal role in maintaining genome integrity, enabling CSC to withstand stressful conditions^44,45^.

Moreover, our study explored the TME modulation by CSC, revealing their impact on tumor immunity. The proximity of Macro_SPP1 to cancer cells suggested potential juxtacrine interactions. Liver CSC, characterized by low proteasome activity and low intracellular ROS levels, were reported to facilitate macrophage migration and recruitment to the tumor site^46^. SPP1+ macrophages were identified as a new marker for the protumor subpopulation of monocyte-derived TAMs^47^. SPP1, a multifunctional secreted phosphorylated glycoprotein, is known for its involvement in cancer cell growth and resistance to chemoradiotherapy, inducing EMT, autophagy, aberrant glucose metabolism, epigenetic alterations, and reduction of drug uptake^48,49^. Inhibition of SPP1 in mice increased T lymphocyte infiltration, suggesting the potential for combined anti-PD-1 therapy with SPP1 inhibition in HCC clinical trials^50^. Macro_SPP1 exhibited elevated expression levels of metalloproteinases, including MMP9, MMP12, and MMP7, capable of degrading ECM proteins, implying their role in chemoresistance and quiescence within the hepatic CSC niche^51,52^. This observation also suggests ECM alterations in the liver CSC niche. Transcription factor HIF1A appeared to potentially regulate both CSC and Macro_SPP1 activities, particularly in hypoxia-induced pathways.

Additionally, we found the co-location of SPP1+macrophages and CSC were related to poor prognosis in HCC. Unraveling the interaction patterns between the two cell types could uncover new therapeutic targets for the combination of CSC-based treatments. Through cell-cell communication analysis, we found SPP1-CD44 as the main ligand-receptor pair involved in the interaction between SPP1+macrophages and CSC. Spatial transcriptomics analysis revealed that the SPP1 signaling pathway was the most activated interaction in the co-location area of CSC and Macro_SPP1. Notably, SPP1-CD44 and SPP1-(ITGAV + ITGB) were the main ligand-receptor pairs involved in cell-cell communication. The SPP1/CD44 axis has been linked to cancer chemoresistance in solid tumors, and it may represent a critical mechanism for cell-to-cell communication between cancer cells and TAMs^53,54^. SPP1 facilitated cancer cell chemoresistance by activating the CD44 receptor, and anti-SPP1 and anti-CD44 antibodies improved cancer cell sensitivity to cisplatin in a mouse model^55^. High CD44 expression has been observed in gastric cancer stem cells, and SPP1-expressing TAMs correlated with a worse clinical outcome in gastric cancer, suggesting the SPP1/CD44 axis may be associated with stemness properties and resistance to anticancer therapies. A similar significance of SPP1/CD44-related signals has been suggested in glioma and HCC^56,57^.

In comparison to prior research, our study holds several strengths. It is the first to comprehensively characterize CSC in HCC and investigate their spatial organization, advancing our understanding of cancer stemness. Additionally, we identified and validated the CSC signature, facilitating CSC detection and targeting. With over 80,000 cells in single-cell and eight spatial transcriptomic samples, our study achieves both breadth and depth. However, certain limitations must be acknowledged. The lack of immunotherapy-treated patients in our cohort necessitates further verification of CSC’ role in immunotherapy. Comprehensive experimental validations are also required to interpret the genes within the CSC signature in the context of the immune response.

## Methods

### Patient samples

Formalin-fixed paraffin-embedded (FFPE) HCC samples were obtained from 32 pre-treatment patients at the Cancer Hospital, Chinese Academy of Medical Science in Beijing, China. The protocol received approval from Institut Curie’s Ethics Committee (No.23/262-4004). Written informed consent was obtained from all patients included in this study. This work was conducted in accordance with all relevant ethical regulations including the Declaration of Helsinki. The American Joint Committee on Cancer (AJCC) stage of each sample was evaluated by pathologists. There were 16 early-stage patients (AJCC I-II) and 16 were advanced-stage patients (AJCC III-IV). Thirty two samples were utilized for IHC, and eight representative samples were employed for spatial transcriptomic sequencing.

### Data and materials

The single-cell data sets of GSE151530 and GSE149614 were acquired from the Gene Expression Omnibus (GEO) database. The corresponding clinical data and metadata were obtained from the original studies. In total, our study encompassed 81,508 cells derived from 45 HCC tumor samples for discovering analysis. In addition, we obtained another two single-cell datasets (GSE166635, GSE146115) for validation.

For bulk-level analysis, we retrieved mRNA expression data and clinical information for HCC patients from TCGA, accessible through UCSC Xena (http://xena.ucsc.edu/). Additionally, we gathered data from 13 publicly available immunotherapy cohorts from the GEO database and original studies for further analysis (Table S2). In the case of the immunotherapy cohorts, patients were categorized into two groups based on their response status: complete response and partial response as responders, and stable disease and progressive disease as non-responders.

### Dimension reduction and clustering analysis

The top 2000 most variable genes were identified using the FindVariableFeatures function and subsequently employed for principal component analysis (PCA) in Seurat package. To mitigate batch effects within the datasets, we applied the Harmony algorithm from the Harmony R package before conducting the clustering analysis. Cell subtypes were then determined using the FindNeighbors and FindCluster functions. We used the curated markers to annotate cells: epithelial cells (EPCAM, KRT8, KRT19), fibroblasts (COL1A1, COL1A2, DCN), endothelial cells (PLVAP, VWF, PECAM1), T cells (CD3D, CD3E, TRAC), B cells (MS4A1, CD79A), myeloid cells (CD14, CD163, CD68, FCGR3A).

### InferCNV for single-cell analysis

We employed the InferCNV analysis (https://github.com/broadinstitute/InferCNV) to identify somatic large-scale chromosomal copy number alterations in each cell. For this purpose, we selected all fibroblasts (*n* = 3722) and endothelial cells (*n* = 4388) as spiked-in controls. To meet the data requirements, we prepared a raw counts matrix, annotation file, and gene/chromosome position file. Next, we created an infercnv object using the CreateInfercnvObject function. Subsequently, the inferCNV analysis was conducted with the default parameters (cutoff=0.1, cluster_by_groups=TRUE, denoise=TRUE, HMM = FALSE).

### CytoTRACE score

The CytoTRACE algorithm is an unsupervised framework used to predict relative differentiation states from single-cell transcriptomes^21^. It has been validated in large-scale datasets and has shown superior performance compared to existing computational techniques for assessing stemness. The CytoTRACE algorithm calculates the gene expression matrix and assigns a score to each individual cell, representing its stemness. In this study, we utilized the R package CytoTRACE to calculate the CytoTRACE score specifically for malignant cells. The ScanoramaCT python module was exploited to mitigate batch effects within the datasets. The CytoTRACE scores range from 0 to 1, with higher scores indicating higher stemness (lower differentiation) and vice versa.

### Differential expression analysis and gene set enrichment analysis

We used FindMarkers in Seurat package with MAST differentially expression analysis method to identify the DEGs between different groups. It was run with cutoff logfc 0.25, only.pos =F. We used GSEA in Rpackage fgsea to determine the enrichment of cancer hallmark and Biological Process Gene Ontology (GO) and Kyoto Encyclopedia of Genes and Genomes (KEGG) genesets.

### Transcription factor analysis

Our objective was to investigate the unique activity of TFs in CSC. TF activity was inferred using the Dorothea resource, which contains signed TF-target interactions (https://saezlab.github.io/dorothea)^23^. To construct TF regulons, we utilized the ‘dorothea regulon human’ wrapper function from the ‘dorothea’ library and selected high-confidence TFs at levels ‘A’, ‘B’, and ‘C’. The run_viper function was then employed to calculate the activities of the regulons. In the context of single-cell data, we constructed regulons based on the mRNA expression levels of each TF and its direct targets. We combined the VIPER algorithm with DoRothEA using the run_viper function to estimate TF activities from the Dorothea regulons.

### Metacell analysis

For metacell analysis, we employed the R metacell package to separate tumor cells into multiple metacells^27^. Initially, we excluded specific mitochondrial genes annotated with the prefix “MT-“, as they are commonly associated with stressed or dying cells. Using gene count matrices, we selected feature genes with a scaled variance (variance/mean on down-sampled matrices) greater than 0.08. These genes were utilized to calculate cell-to-cell similarity using Pearson correlations. We constructed balanced K-nn similarity graphs for malignant cells, where the parameter K was set to 100 and limited the number of neighbors for each cell. Subsequently, we performed resampling procedures, resampling 75% of the cells with 500 iterations, and executed coclustering graph construction with a minimum cluster size of 50. The expression of each gene in a specific metacell was defined as the average expression of that gene in the corresponding individual cells. Additionally, the CytoTRACE score for each metacell was calculated as the mean score of its constituent individual cells.

### WGCNA

WGCNA was performed using the R package WGCNA to identify genes associated with CSC^58^. The pickSoftThreshold function was utilized to determine appropriate weighting parameters for nearby components, which served as a soft threshold for network construction. Subsequently, a weighted adjacency matrix was generated, and gene modules were constructed using hierarchical clustering based on the 1-Tom dissimilarity measure of the topological overlap matrix. Finally, the correlation between the modules and the CytoTRACE score was calculated. In our specific analysis, the initial step involved the inclusion of expression profiles from the top 5000 variable genes identified within the 320 metacells. Hierarchical clustering was then applied to these 5000 genes, resulting in their division into 27 distinct modules. Regarding the relation of modules to the metacells, we first calculated the CytoTRACE score for each metacell as the mean score derived from its constituent individual cells. The module eigengene, defined as the first principal component of the expression matrix represents the module expressions of the module. The eigengene can be thought of as a weighted average expression profile. Subsequently, the correlation between the eigengene in all modules and the CytoTRACE score was determined using a univariate regression model to assess the relationship between the gene expression patterns within these modules and the stemness characteristics represented by the CytoTRACE scores.

### AUCell

Initially, for each cell, we utilized an expression matrix to compute gene expression rankings employing the AUCell_buildRankings function, using the default parameters provided by the package. The AUCell_calcAUC function was employed to compute the area-under-the-curve (AUC) values based on the gene expression rankings. These AUC values essentially represent the fraction of CSC signature genes positioned within the top-ranking genes for each cell. Cells with heightened expression levels for genes within the CSC signature were associated with correspondingly elevated AUC values, signifying a stronger association with the CSC characteristics.

### Survival analysis

Survival analysis was conducted using the R package survival. The Cox proportional hazards model was employed to calculate the hazard ratio with a reported 95% confidence interval, and Kaplan-Meier survival curves were generated using the survfit function. The “maxstat.test” function from the R package maxstat was employed to perform dichotomy of cell population infiltration or gene expression by testing all potential cutting points to identify the maximum rank statistic. Based on the selected maximum logarithm statistics, patients were divided into two groups. The two-sided log-rank test was then applied to compare the Kaplan-Meier survival curves. The comparison of the percentage of patients who responded to immunotherapy treatment between different groups was determined using the Chi-squared test.

### IHC analysis

A total of 32 HCC samples were subjected to IHC analysis. The histologic stage of all HCC tissues were determined by the pathology department. The slices were then dewaxed and incubated with specific primary antibodies (ENO1: ab227978, TKT: ab131331, CD63: ab134045, G6PD: ab133525 and KRT8: ab53280) at 4 C overnight, followed by incubation with biotinylated secondary antibody (Proteintech, Wuhan, China) at room temperature for 1 hour. Positive staining was detected using DAB chromogenic reagent, and each section was counterstained with hematoxylin. Each sample was assigned a score according to the intensity of the staining (0 = no staining; 1 = weak staining; 2 = moderate staining; and 3 = strong staining) and the proportion of stained cells (0 = 0%; 1 = 1–25%; 2 = 25–50%; 3 = 50–75%; 4 = 75–100%). The final score was calculated as the staining intensity multiplying positive area score, ranging from 0 to 12. The IHC results of tissues were independently reviewed by two experienced pathologists who were blinded to the clinical parameters.

### Spatial transcriptomics sequencing

#### Sample fixation and H&E staining

Eight FFPE human cancer tissue blocks were obtained from patients with HCC. Five-micrometer FFPE sections of these samples were placed on IHC slides. The slides were then incubated at 42 °C for 2 hours and allowed to dry at room temperature. Afterward, the slides were dried again for 3 hours at 60 °C. Hematoxylin (Dako, Part number S330930-2) and Eosin (Sigma-Aldrich, Product number HT110216) were used for H&E staining, with the staining times adjusted according to the tissue type. Approximately 100 µL of 85% Glycerol (Thermofisher, Catalog number 15514011) was added, coverslips were applied, and tissue imaging was performed. To remove the coverslips, a beaker filled with Milli-Q water was used.

#### Probe hybridization

The Visium slide was placed into a cassette. 100 µL of 0.1 N HCl (Sigma-Aldrich, Product number H1758) was added to each well and incubated at 42 °C for 15 minutes. The HCl was then removed, and decrosslinking buffer was added. The slide was incubated at 95 °C for 1 hour. The Pre-hybridization step was continued according to The Visium Spatial Gene Expression for FFPE reagent kit (10×Genomics, User Guide CG000407 Rev C, human transcriptome Product number 1000338). 100 µL of Pre-hybridization mix was added to each well and incubated at room temperature for 15 minutes. After incubation, the Pre-hybridization mix was removed, and 100 µL of Hybridization mix was added. The Visium slide was incubated with the Hybridization mix overnight at 50 °C.

#### Probe ligation, probe release and extension, probe elution, and library preparation

For the remaining steps of library preparation, including probe ligation, probe release and extension, probe elution, and FFPE library construction, the user guide of “Visium Spatial Gene Expression for FFPE reagent kit” (10× Genomics, User Guide CG000407 Rev C, mouse transcriptome Product number 1000339, human transcriptome Product number 1000338) was followed. The finished libraries were sequenced on Novaseq6000 (Illumina). The length of read 1 and read 2 were 28 base pairs and 91 base pairs, respectively.

### Pathological annotations for HE images

All Visium spots in the Visium sections were annotated individually by two pathologists, Lin Li and Tongji Xie. Using a cell-type-specific coverage threshold of >50%, the pathologists annotated the spots according to histological classes, including normal hepatocytes, tumor cells, stromal cells, and immune cells.

### Clustering analysis of spatial transcriptomics

The gene-spot matrices generated from the ST data were analyzed using the Seurat package in R and stlearn in Python. For stlearn, the input consisted of the expression matrix of the ST data and the HE-stained histological image. The HE-stained histological images were extracted and converted to a resolution of 2048 pixels using ResNet, a well-established convolutional neural network model commonly used for image classification in computer vision, and ImageNet, a dataset containing millions of images. PCA was performed to reduce the spatial gene expression and pixel matrices to 50 principal components each. The gene expression was then adjusted morphologically using the SME normalization algorithm from stlearn based on the spot image matrix, resulting in a morphologically adjusted gene expression matrix. Additionally, FindVariableFeatures, FindNeighbors, and FindCluster functions were employed for clustering analysis in each sample.

### Identification of malignant cells in spatial analysis

A series of immune-related signatures, including pan-immune markers (PTPRC), pan-T cell markers (CD2, CD3D, CD3E, CD3G), B cell markers (CD79A, MS4A1, CD79B), and myeloid cell markers (CD68, CD14), were utilized for spot scoring. The average value of these features was designated as the normal tissue expression score (NormalScore) for each spot. Based on the clustering results, the cluster with the highest median NormalScore was defined as the inferCNV reference. Subsequently, the inferCNV analysis was conducted with the following parameters: cutoff = 0.1, cluster_by_groups = FALSE, denoise = TRUE, HMM = TRUE, analysis_mode = “subclusters”, tumor_subcluster_partition_method = “random_trees”, HMM_type = “i6”. The Hidden Markov Model (HMM) was employed to assess the CNV level for the spots. To achieve a more accurate classification of spatial spots and distinguish malignant spots from non-malignant spots, hierarchical clustering based on tree partitioning using the inferCNV package with the random trees method was performed, dividing all spots into 8 clusters. Reference spots were labeled as “reference”. In the context of the inferCNV analysis, a gene state of 3 indicated no CNV variation, a state greater than 3 indicated CNV amplification, and a state less than 3 indicated CNV deletion. The CNV score of each gene was defined as the absolute value of the gene state minus 3. The CNV score of each cluster was calculated as the sum of the CNV scores of all genes. The tumor cluster was defined based on the CNV score and the pathological annotations.

### Cell-Cell communication analysis

CellChat, an R package, was utilized to analyze communication interactions and identify communicating molecules at the single-cell level between CSC and other principal cell types. CellChatDB.human was employed to analyze the primary signaling inputs and outputs among all cell clusters. The role of CSC in the cell-cell communication network was determined using the netAnalysis_signalingRole_scatter function. The aggregated cell-cell communication network was calculated using the “aggregateNet” function in CellChat, and the signaling from each cell group was visualized. Signaling groups based on functional or structural similarity were identified using the “computeNetSimilarity” function. The outgoing/incoming signaling patterns of cells were calculated using the “netAnalysis_signalingRole_heatmap” function. Ligand-receptor pairs involved in signaling between cells were identified using the “netVisual_bubble” function.

### Statistical analysis

The Mann-Whitney U test was performed to analyze the differences between the two groups. Spearman’s correlation test was used to assess the correlations between two variables. A two-tailed P-value of 0.05 was considered statistically significant. R 4.2.0 was used for the entire data processing, statistical analysis, and plotting procedures.

### Reporting summary

Further information on research design is available in the Nature Research Reporting Summary linked to this article.

### Supplementary information

REPORTING SUMMARY

supplementary materials

## Data Availability

The names of the public accessible repositories and accession numbers can be found in Table S4. The raw sequence data reported in this paper have been deposited in the Genome Sequence Archive in National Genomics Data Center, China National Center for Bioinformation/Beijing Institute of Genomics, Chinese Academy of Sciences (GSA: HRA006757) and was available upon request.

## Code availability

This study utilized publicly available packages with R version 4.2.0. The code used for data analysis and figure generation presented in this publication can be obtained upon request.
