# Supplementary material for: Single-cell and spatial analyses revealed the co-location of cancer stem cells and SPP1+ macrophage in hypoxic region that determines the poor prognosis in hepatocellular carcinoma
Source: NPJ Precis Oncol. 2024 Mar 23;8:75. doi: 10.1038/s41698-024-00564-3 (PMC10960828; doi:10.1038/s41698-024-00564-3)
Supplement: Supplementary file 1 — REPORTING SUMMARY [file 41698_2024_564_MOESM1_ESM.pdf]

nature portfolio

Corresponding author(s): NPJPRECISIONONCOLOGY-04054R

Last updated by author(s): Nov 23, 2023

# Reporting Summary

Nature Portfolio wishes to improve the reproducibility of the work that we publish. This form provides structure for consistency and transparency in reporting. For further information on Nature Portfolio policies, see our Editorial Policies and the Editorial Policy Checklist.

## Statistics

For all statistical analyses, confirm that the following items are present in the figure legend, table legend, main text, or Methods section.

| n/a | Confirmed | |
|---|---|---|
| ☐ | ☒ | The exact sample size (*n*) for each experimental group/condition, given as a discrete number and unit of measurement |
| ☐ | ☒ | A statement on whether measurements were taken from distinct samples or whether the same sample was measured repeatedly |
| ☐ | ☒ | The statistical test(s) used AND whether they are one- or two-sided<br>*Only common tests should be described solely by name; describe more complex techniques in the Methods section.* |
| ☒ | ☐ | A description of all covariates tested |
| ☒ | ☐ | A description of any assumptions or corrections, such as tests of normality and adjustment for multiple comparisons |
| ☐ | ☒ | A full description of the statistical parameters including central tendency (e.g. means) or other basic estimates (e.g. regression coefficient) AND variation (e.g. standard deviation) or associated estimates of uncertainty (e.g. confidence intervals) |
| ☐ | ☒ | For null hypothesis testing, the test statistic (e.g. *F*, *t*, *r*) with confidence intervals, effect sizes, degrees of freedom and *P* value noted<br>*Give P values as exact values whenever suitable.* |
| ☒ | ☐ | For Bayesian analysis, information on the choice of priors and Markov chain Monte Carlo settings |
| ☒ | ☐ | For hierarchical and complex designs, identification of the appropriate level for tests and full reporting of outcomes |
| ☒ | ☐ | Estimates of effect sizes (e.g. Cohen's *d*, Pearson's *r*), indicating how they were calculated |

*Our web collection on statistics for biologists contains articles on many of the points above.*

## Software and code

Policy information about availability of computer code

| | |
|---|---|
| Data collection | No software was used in the data collection process. |
| Data analysis | R 4.2.0 was used for the entire data processing, statistical analysis, and plotting procedures. |

For manuscripts utilizing custom algorithms or software that are central to the research but not yet described in published literature, software must be made available to editors and reviewers. We strongly encourage code deposition in a community repository (e.g. GitHub). See the Nature Portfolio guidelines for submitting code & software for further information.

## Data

Policy information about availability of data

All manuscripts must include a data availability statement. This statement should provide the following information, where applicable:

- Accession codes, unique identifiers, or web links for publicly available datasets
- A description of any restrictions on data availability
- For clinical datasets or third party data, please ensure that the statement adheres to our policy

The names of the public accessible repositories and accession numbers can be found in Table S4. The spatial transcriptomics data in our study is available upon request.

## Research involving human participants, their data, or biological material

Policy information about studies with human participants or human data. See also policy information about sex, gender (identity/presentation), and sexual orientation and race, ethnicity and racism.

| | |
|---|---|
| Reporting on sex and gender | The breakdown of M vs. F sex among the cohort was reported. No additional sex-specific analyses were performed. |
| Reporting on race, ethnicity, or other socially relevant groupings | N/A. No race or ethnicity information was available. |
| Population characteristics | The clinical information incorporated age, sex, viral hepatitis status, and pathological stage (AJCC staging system) was in table S3. |
| Recruitment | Formalin-fixed paraffin-embedded (FFPE) HCC samples were obtained from 32 pre-treatment patients at the Cancer Hospital, Chinese Academy of Medical Science in Beijing, China, following institutional ethical guidelines and after obtaining informed consent from patients. |
| Ethics oversight | The protocol received approval from Institut Curie's Ethics Committee (No.23/262-4004). |

Note that full information on the approval of the study protocol must also be provided in the manuscript.

# Field-specific reporting

Please select the one below that is the best fit for your research. If you are not sure, read the appropriate sections before making your selection.

☒ Life sciences ☐ Behavioural & social sciences ☐ Ecological, evolutionary & environmental sciences

For a reference copy of the document with all sections, see nature.com/documents/nr-reporting-summary-flat.pdf

# Life sciences study design

All studies must disclose on these points even when the disclosure is negative.

| | |
|---|---|
| Sample size | Formalin-fixed paraffin-embedded (FFPE) HCC samples were obtained from 32 pre-treatment patients at the Cancer Hospital, Chinese Academy of Medical Science in Beijing, China, following institutional ethical guidelines and after obtaining informed consent from patients. |
| Data exclusions | 25 samples were excluded from further analysis due to a low number of malignant cells (n<100), resulting a total of 56,022 cells from 20 samples for further analysis, |
| Replication | To validate the signature, we recruited two additional single-cell datasets of HCC (GSE166635, GSE146115), and the correlation between the two scores across two datasets was 0.88 and 0.81, respectively. In addition, we incorporated two stemness-related signatures from prominent databases (KEGG and REACTOME), which could be found in table S2. These gene sets were then utilized to assess and cross-validate the identified CSC signature across three datasets. We calculated the scores for these curated signatures and observed a significantly positive correlation between each signature and our identified CSC score across all analyzed datasets. |
| Randomization | N/A. Randomization was not performed since grouping was performed based on tumor-location. This is a retrospective study. |
| Blinding | N/A Blinding was not performed. This is a retrospective study |

# Reporting for specific materials, systems and methods

We require information from authors about some types of materials, experimental systems and methods used in many studies. Here, indicate whether each material, system or method listed is relevant to your study. If you are not sure if a list item applies to your research, read the appropriate section before selecting a response.

### Materials & experimental systems

| n/a | Involved in the study | |
|---|---|---|
| ☐ | ☒ | Antibodies |
| ☒ | ☐ | Eukaryotic cell lines |
| ☒ | ☐ | Palaeontology and archaeology |
| ☒ | ☐ | Animals and other organisms |
| ☒ | ☐ | Clinical data |
| ☒ | ☐ | Dual use research of concern |
| ☒ | ☐ | Plants |

### Methods

| n/a | Involved in the study | |
|---|---|---|
| ☒ | ☐ | ChIP-seq |
| ☒ | ☐ | Flow cytometry |
| ☒ | ☐ | MRI-based neuroimaging |

## Antibodies

| | |
|---|---|
| Antibodies used | all antibodies were obatined from abcam, including ENO1: ab227978, TKT: ab131331, CD63: ab134045, G6PD: ab133525 and KRT8: ab53280 |
| Validation | The slices were then dewaxed and incubated with specific primary antibodies (ENO1: ab227978, TKT: ab131331, CD63: ab134045, G6PD: ab133525 and KRT8: ab53280) at 4C overnight, followed by incubation with biotinylated secondary antibody (Proteintech, Wuhan, China) at room temperature for 1 hour, as stated in the methods. |

## Plants

| | |
|---|---|
| Seed stocks | *Report on the source of all seed stocks or other plant material used. If applicable, state the seed stock centre and catalogue number. If plant specimens were collected from the field, describe the collection location, date and sampling procedures.* |
| Novel plant genotypes | *Describe the methods by which all novel plant genotypes were produced. This includes those generated by transgenic approaches, gene editing, chemical/radiation-based mutagenesis and hybridization. For transgenic lines, describe the transformation method, the number of independent lines analyzed and the generation upon which experiments were performed. For gene-edited lines, describe the editor used, the endogenous sequence targeted for editing, the targeting guide RNA sequence (if applicable) and how the editor was applied.* |
| Authentication | *Describe any authentication procedures for each seed stock used or novel genotype generated. Describe any experiments used to assess the effect of a mutation and, where applicable, how potential secondary effects (e.g. second site T-DNA insertions, mosiacism, off-target gene editing) were examined.* |
