# Supplementary material for: Single-cell and spatial analyses revealed the co-location of cancer stem cells and SPP1+ macrophage in hypoxic region that determines the poor prognosis in hepatocellular carcinoma
Source: NPJ Precis Oncol. 2024 Mar 23;8:75. doi: 10.1038/s41698-024-00564-3 (PMC10960828; doi:10.1038/s41698-024-00564-3)
Supplement: Supplementary file 2 — supplementary materials [file 41698_2024_564_MOESM2_ESM.pdf]

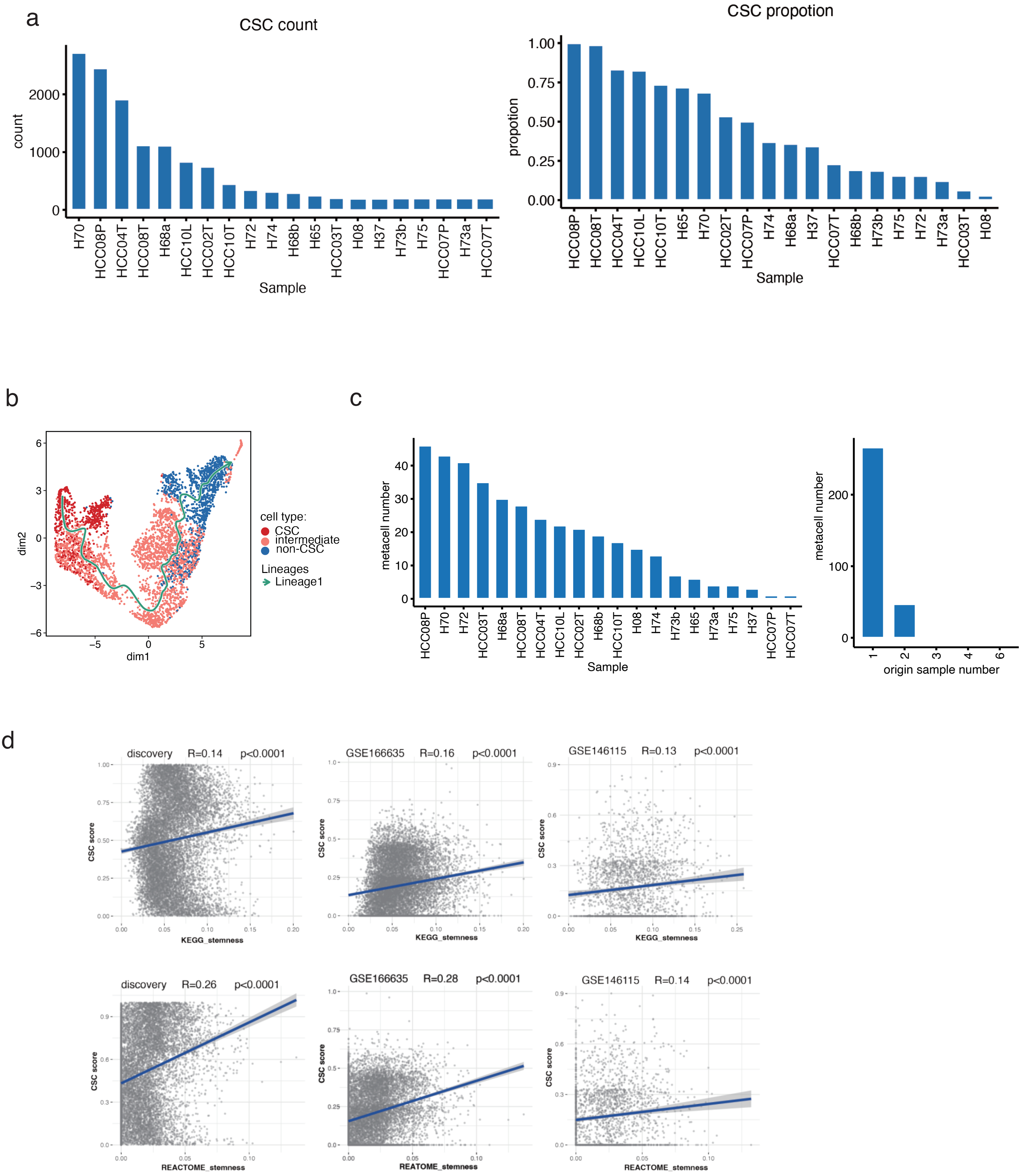

Figure S1: The landscape of CSC in the single-cell data. (a) The cell number and proportion of CSC in each sample in the discovery cohort. (b) The pseudotime trajectory of CSC, non-CSC and the intermediate state. (c)The relationship between the 320 metacells and the original sample (d) The scores of two stemness-related signatures from prominent databases (KEGG and REACTOME) had a significantly positive correlation with our identified CSC score.

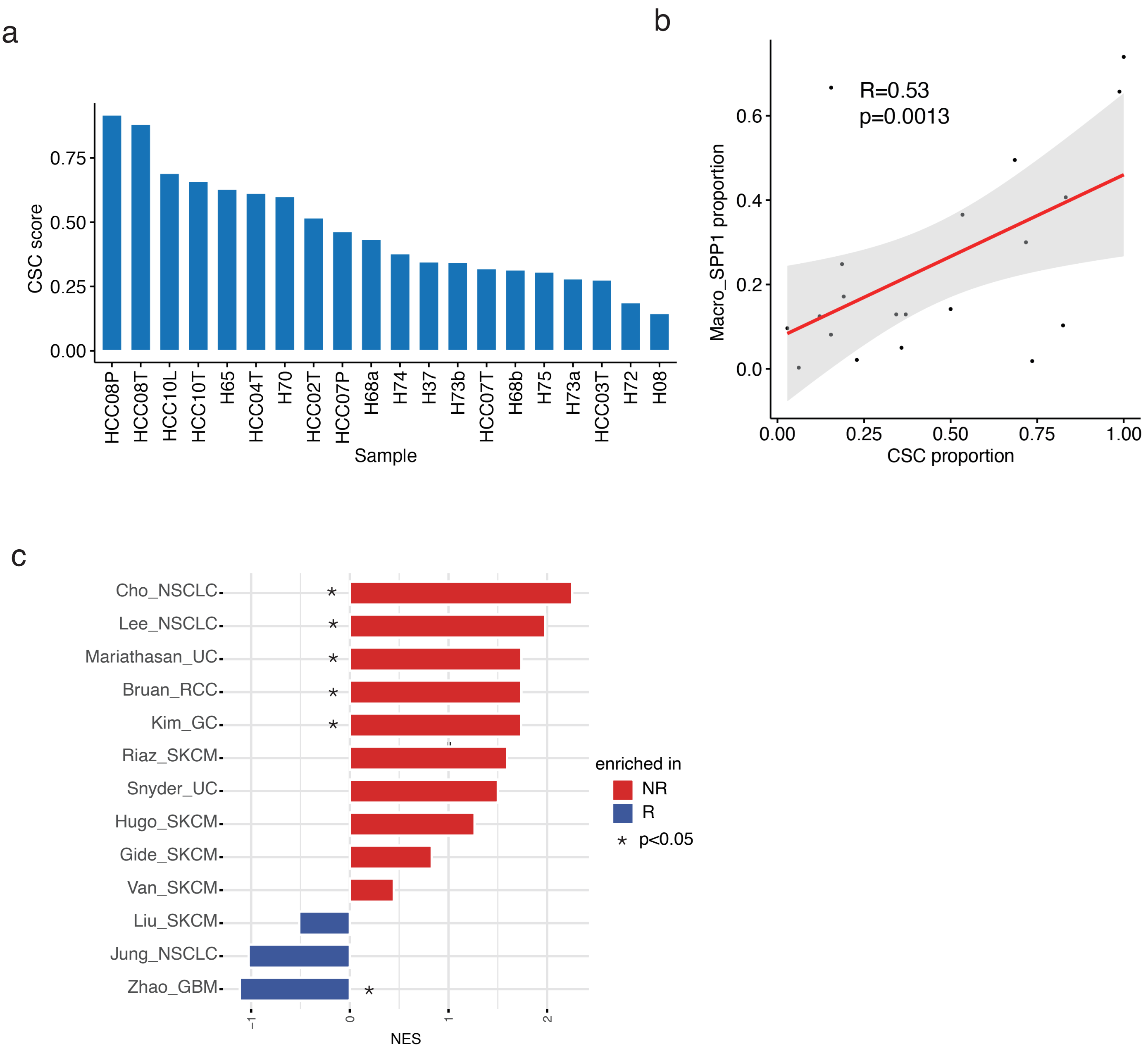

Figure S2: The investigation between CSC and Macro_SPP1. (a) The CSC score per sample. (b) The positive correlation in cell counts between CSC and Macro_SPP1. (c) Pathway enrichment analysis revealing that Macro_SPP1 was enriched in non-responders in multiple immunotherapy cohorts.

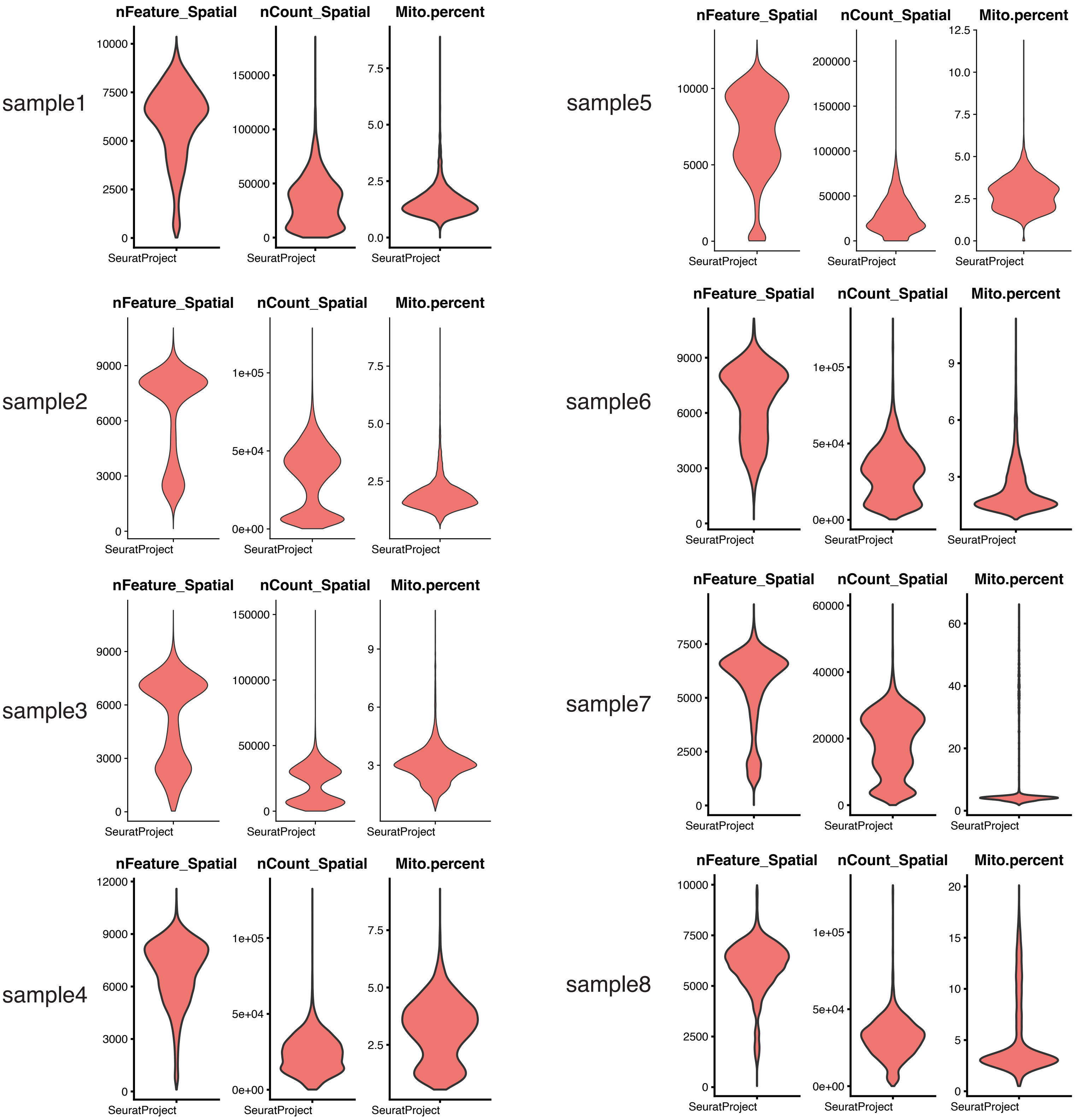

Figure S3: The violin plots of nFeature, nCount, and the mitochondrial gene percentage of eight ST samples.

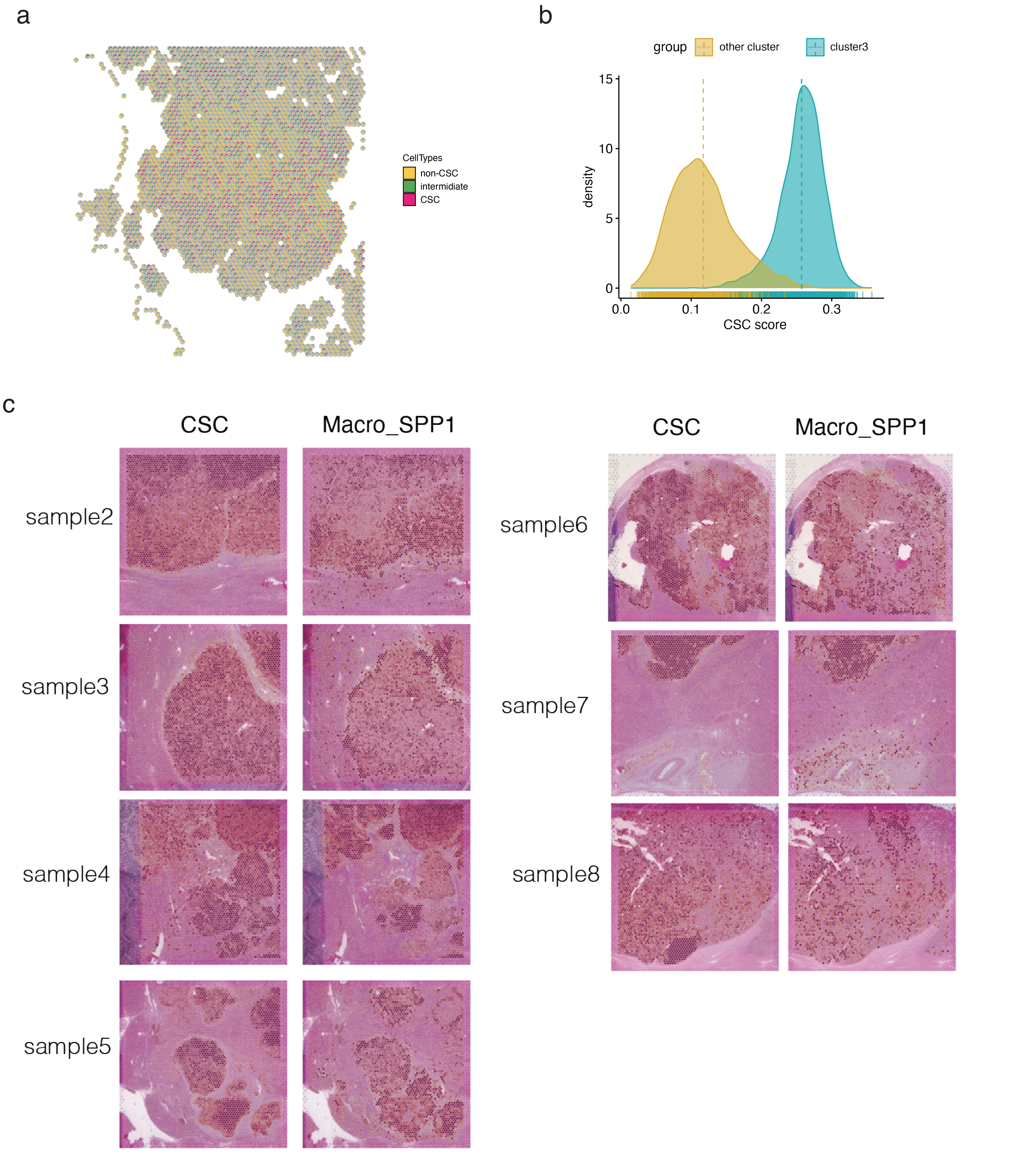

Figure S4: The spatial patterns of CSC and Macro_SPP1 in ST samples. (a) The cell type deconvolution analysis using CARD revealed that CSC mainly co-located with other cell types within each spot of cluster 3. (b) The density plot displayed that majority of spots in the cluster 3 had higher CSC score than other clusters and had relatively close CSC score. (c) The feature plots revealed the co-location between CSC and Macro_SPP1 in ST samples.

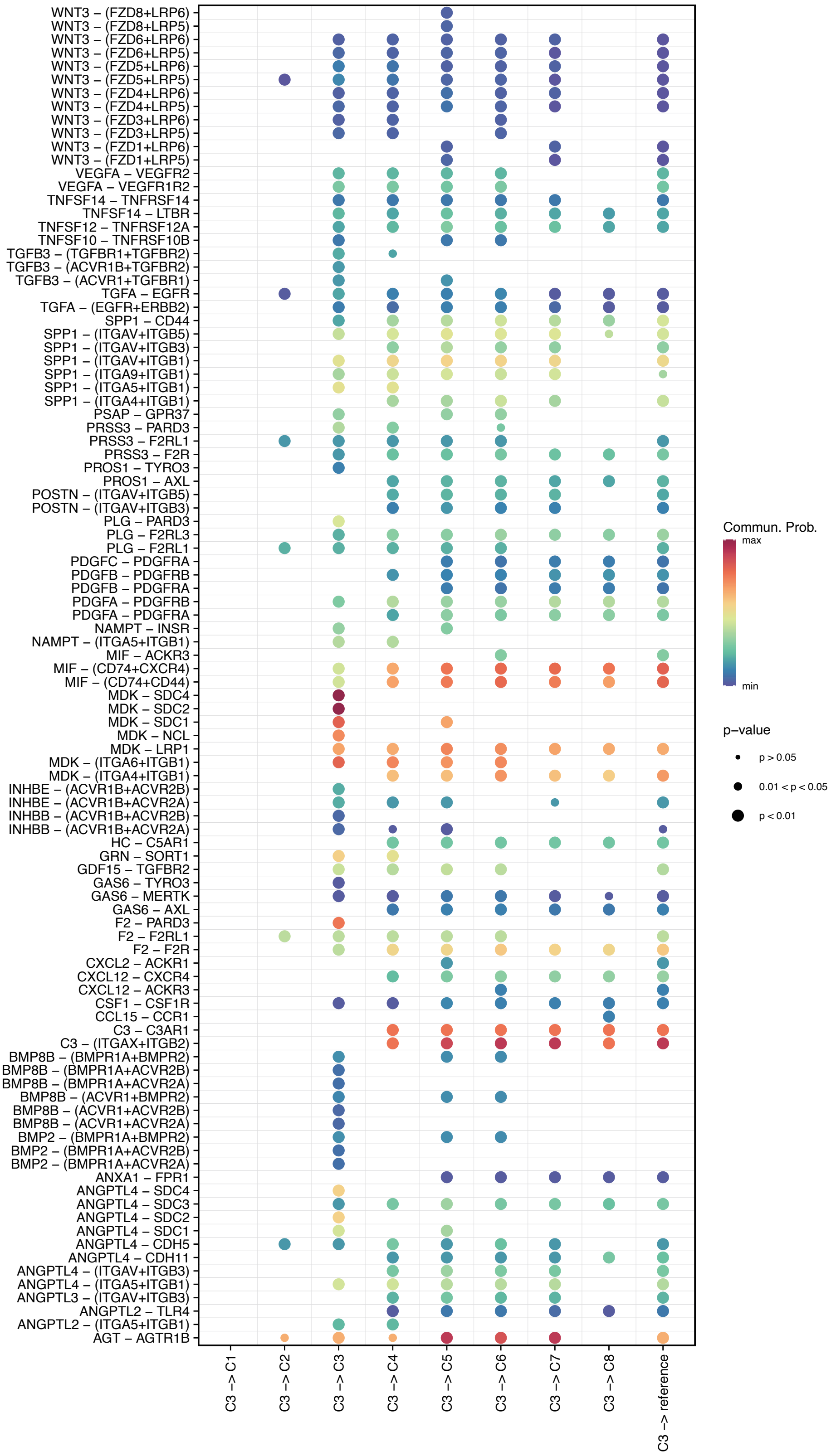

Figure S5: The ligand-receptor pairs signaled out from cluster 3 to all clusters.

Table S1. The list of genes in the CSC signature.

ENO1	HSPB1	GLO1
TKT	PON1	PTP4A1
KRT8	POLR2J	PERP
CD63	PRDX4	KDELR2
G6PD	GPC3	MALSU1
GSTM3	DEFB1	CYCS
S100A13	LAPTM4B	GGCT
CCT3	POLR2K	MPLKIP
PFDN2	ENY2	PPIA
ELF3	MRPL13	SEC61G
SNRPE	SQLE	CCT6A
COA6	TSTA3	CHCHD2
COX20	PUF60	
ANXA4	STOML2	
NDUFB3	CLTA	
CMTM8	LDHA	
GLB1	CCDC34	
SPCS1	MDK	
ALB	COA4	
CCT5	ATP5C1	
SEPP1	NUDT5	
HMGCS1	PRDX3	
VDAC1	BNIP3	
NHP2	GAPDH	
TUBB2A	TPI1	
MRPS18B	TMEM106C	
TUBB	CDK4	
PFDN6	TSPAN8	
CUTA	PSMB5	
HMGA1	GPX2	
SNRPC	CKB	

Table S2. Two stemness-related signatures from databases (KEGG and REACTOME).

REACTOME	KEGG				
CDX2	ACVR1	FGFR2	INHBE	OTX1	SMARCA
DKK1	ACVR1B	FGFR3	ISL1	PAX6	SOX2
EOMES	ACVR1C	FGFR4	JAK1	PCGF1	STAT3
EPAS1	ACVR2A	FZD1	JAK2	PCGF2	TBX3
EPHA1	ACVR2B	FZD10	JAK3	PCGF3	TCF3
FGF2	AKT1	FZD2	JARID2	PCGF5	WNT1
FOXD3	AKT2	FZD3	KLF4	PCGF6	WNT10A
GATA6	AKT3	FZD4	KRAS	PIK3CA	WNT10B
GSC	APC	FZD6	LHX5	PIK3CB	WNT11
HHEX	AXIN1	FZD7	LIF	PIK3CD	WNT16
HIF3A	AXIN2	FZD8	LIFR	PIK3CG	WNT2
KLF4	BMI1	FZD9	MAP2K1	PIK3R1	WNT3
LIN28	BMP2	GRB2	MAP2K2	PIK3R2	WNT4
NANOG	BMP4	GSK3B	MAPK1	PIK3R3	WNT5A
NR5A1	BMPR1A	HAND1	MAPK11	PIK3R5	WNT5B
NR6A1	BMPR1B	HESX1	MAPK12	POU5F1	WNT6
PBX1	BMPR2	HNF1A	MAPK13	POU5F1B	WNT7A
POU5F1	C2orf31	HRAS	MAPK14	RAF1	WNT7B
PRDM14	CTNNB1	ID1	MAPK3	REST	WNT8A
SALL4	DLX5	ID2	MEIS1	RIF1	WNT8B
SMAD2	DUSP9	ID3	MYC	SETDB1	WNT9A
SMAD4	DVL1	ID4	MYF5	SKIL	WNT9B
SOX2	DVL2	IGF1	MYST3	SMAD1	ZFHX3
STAT3	DVL3	IGF1R	NANOG	SMAD2	ZIC3
TDGF1	ESRRB	IL6ST	NEUROG1	SMAD3	
TSC22D1	ESX1	INHBA	NODAL	SMAD4	
ZIC3	FGF2	INHBB	NRAS	SMAD5	
ZSCAN10	FGFR1	INHBC	ONECUT1	SMAD9	

Table S3.the clinical information of our in-house cohort and the single-cell cohort

	**Single-cell cohort (16 patients)**	**In-house cohort (32 patients)**	
**Factor**	**Frequency (proportion)**	**Frequency (proportion)**	**P value**
**Age (years)**			0.6788
<65	6 (375%)	14 (43.75%)	
≥65	10 (62.5%)	18 (56.25%)	
**Sex**			0.3148
Male	14 (87.5%)	24 (75.0%)	
Female	2 (12.5%)	8 (25.0%)	
**Viral hepatitis status**			0.2733
Positive	12 (75.0%)	28 (87.5%)	
Negative	4 (25.0%)	4 (12.5%)	
**Pathological stage (AJCC staging system)**			0.1932
I/II	4 (25.0%)	16 (50.0%)	
III/IV	12 (75.0%)	16 (50.0%)	

Table S4: The list of datasets used in this study.

No.	Dataset	Source	Sample Type	Number and description of samples	PubMed ID
Single cell RNA seq cohorts					
1	GSE151530	Ma et al.	HCC	32 HCC samples	PMID: 34216724
2	GSE149614	Lu et.al	HCC	13 HCC samples	PMID: 35933472
3	GSE166635	Meng et.al	HCC	2 HCC samples	PMID: 33619115
4	GSE146115	Su et.al	HCC	4 HCC samples	PMID: 33531041
Bulk RNA-seq dataset of HCC cohorts					
5	TCGA_LIHC	TCGA	HCC	424 HCC samples	PMID: 24071849
Immunotherapy cohorts					
6	Cho_NSCLC	Cho et al.	non-small-cell lung cancer	16 NSCLC patients treated anti-PD-1	PMID: 32879421
7	Lee_NSCLC	Lee et al.	non-small-cell lung cancer	22 LUAD patients treated anti-PD-1	PMID: 33857424
8	Jung_NSCLC	Jung et al.	non-small-cell lung cancer	27 NSCLC patients treated anti-PD-1	PMID: 32762727
9	Riaz_SKCM	Riaz et al.	Melanoma	49 melanoma patients treated anti-PD-1	PMID: 29033130
10	Liu_SKCM	Liu, D. et al.	Melanoma	121 melanoma patients treated anti-PD-1	PMID: 31792460
11	Gide_SKCM	Gide et al.	Melanoma	73 melanoma patients treated immunotherapy	PMID: 30753825
12	Hugo_SKCM	Hugo et.al	Melanoma	28 patients with pre-anti-PD-1 therapy	PMID: 26997480
13	Mariathasan_BLCA	Mariathasan et al.	Bladder urothelial carcinoma	348 patients with anti-PD-1 therapy	PMID: 32416780
14	Braun_RCC	Braun et al.	Renal cell carcinoma	181 patients with immunotherapy	PMID: 32472114
15	Zhao_GBM	Zhao et al.	Glioblastoma	17 patients with immunotherapy	PMID: 28912897
16	Van_SKCM	Van et al.	Melanoma	36 patients with immunotherapy	PMID: 27081077
17	Kim_GC	Kim et al.	Gastric cancer	45 patients with immunotherapy	PMID: 30013197
18	Snyder_UC	Snyder et al.	Urothelial carcinoma	25 patients with immunotherapy	PMID: 28552987

Table S5: The list of marker genes of Macro_SPP1.

gene	p_val	avg_log2FC	pct.1	pct.2	p_val_adj
MMP9	0	2.552617839	0.781	0.28	0
MMP12	1.2193E-182	2.343360576	0.515	0.158	3.1392E-178
SPP1	4.1811E-232	1.882777389	0.976	0.718	1.0765E-227
PLIN2	5.5484E-296	1.756183355	0.982	0.729	1.4285E-291
IFI6	1.0317E-285	1.546025374	0.916	0.543	2.6564E-281
ADM	3.8174E-218	1.526216498	0.702	0.275	9.8288E-214
VCX3A	0	1.444100749	0.61	0.033	0
FABP5	3.9059E-181	1.39191582	0.948	0.728	1.0057E-176
CD36	1.2522E-265	1.387484502	0.681	0.226	3.2239E-261
CSTB	7.2461E-216	1.337108955	0.995	0.914	1.8657E-211
FCGR2B	1.6039E-147	1.298319384	0.631	0.299	4.1294E-143
CD52	3.8256E-130	1.291056121	0.821	0.505	9.8498E-126
CKB	0	1.281496004	0.654	0.089	0
CXCL3	1.27089E-96	1.270827239	0.755	0.432	3.27217E-92
RP11-1143G9.4	3.64479E-100	1.263744244	0.577	0.286	9.38425E-96
CTSL	8.2681E-160	1.220779044	0.943	0.704	2.1288E-155
RGCC	2.9737E-131	1.213930919	0.731	0.41	7.6564E-127
MMP7	5.0492E-116	1.202328523	0.13	0.014	1.3E-111
MDK	3.1984E-269	1.201661945	0.725	0.274	8.235E-265
HSPA6	1.0442E-220	1.199345025	0.837	0.373	2.6886E-216
SDC2	8.5283E-130	1.167850793	0.585	0.285	2.1958E-125
NUPR1	1.60655E-92	1.142765275	0.776	0.549	4.13639E-88
CCL20	6.74661E-75	1.119691691	0.607	0.322	1.73705E-70
CCL7	1.2334E-142	1.116283361	0.225	0.037	3.1757E-138
CD9	1.96E-219	1.105947523	0.901	0.482	5.0465E-215
NQO1	0	1.104210425	0.654	0.146	0
CXCL2	3.53771E-72	1.065537458	0.777	0.527	9.10855E-68
GPNMB	1.3968E-172	1.04989311	0.95	0.65	3.5963E-168
FABP4	1.4649E-103	1.015597858	0.221	0.049	3.77172E-99
PAGE5	0	1.014276498	0.546	0.084	0
MT1G	5.683E-185	0.99340212	0.666	0.246	1.4632E-180
ANGPTL4	3.0891E-151	0.962943137	0.39	0.109	7.9534E-147
NFKBIA	3.8626E-105	0.960023821	0.963	0.834	9.945E-101
HEXB	8.2211E-148	0.936805039	0.81	0.521	2.1167E-143
CXCL8	1.12748E-74	0.934431936	0.832	0.57	2.90292E-70
FTL	8.9949E-213	0.923038702	1	1	2.3159E-208
ANXA1	1.7575E-104	0.915432188	0.901	0.678	4.52509E-100

ACP5	5.6482E-106	0.913894354	0.793	0.516	1.4543E-101
FTH1	6.6984E-211	0.912674135	1	0.997	1.7246E-206
CCL3L3	6.803E-75	0.908860759	0.819	0.543	1.75157E-70
PLD3	6.2556E-106	0.905521699	0.868	0.635	1.6106E-101
S100A10	2.1905E-175	0.899683527	0.986	0.853	5.6398E-171
HSPB1	7.0718E-172	0.897349281	0.975	0.827	1.8208E-167
MIF	6.4383E-173	0.89713106	0.969	0.723	1.6577E-168
PHLDA1	4.38187E-74	0.879795173	0.558	0.315	1.1282E-69
MT1H	7.2984E-141	0.875381866	0.39	0.106	1.8791E-136
ANXA2	6.0861E-178	0.871199635	0.973	0.842	1.567E-173
PPP1R15A	5.2116E-114	0.866664535	0.88	0.654	1.3418E-109
BNIP3	4.8129E-146	0.862915487	0.741	0.394	1.2392E-141
NGFRAP1	1.8247E-164	0.851509923	0.704	0.325	4.6982E-160
ZFAND2A	5.4523E-179	0.84954724	0.765	0.358	1.4038E-174
SLC2A1	6.9401E-145	0.849482646	0.408	0.125	1.7869E-140
LY6E	1.5614E-147	0.835988306	0.871	0.589	4.02E-143
GLB1	1.177E-170	0.831305036	0.6	0.253	3.0305E-166
JUN	3.27859E-84	0.829368547	0.89	0.668	8.44138E-80
CD68	1.4426E-166	0.825437868	0.982	0.834	3.7143E-162
ERO1A	1.33716E-98	0.813142661	0.632	0.359	3.44278E-94
MT1X	1.9824E-110	0.808294378	0.837	0.518	5.1042E-106
MT1F	4.51708E-91	0.804893823	0.48	0.214	1.16301E-86
DDIT4	6.388E-75	0.803131684	0.714	0.45	1.64472E-70
CCL3	1.26227E-51	0.798581692	0.899	0.721	3.24997E-47
DEFB1	5.1788E-281	0.73694293	0.525	0.111	1.3334E-276
NDUFA4L2	0	0.731901845	0.415	0.053	0
EIF4A3	4.84695E-65	0.710966124	0.717	0.494	1.24795E-60
SERPINH1	5.0765E-123	0.697024268	0.466	0.174	1.307E-118
KRT10	6.4855E-127	0.677397802	0.714	0.381	1.6698E-122
PCOLCE2	1.7061E-204	0.659751418	0.3	0.048	4.3927E-200
ITGB2	3.07459E-89	0.657674937	0.915	0.71	7.91616E-85
HSPA1B	1.1261E-103	0.65518283	0.894	0.614	2.8993E-99
SDC4	6.78489E-100	0.652694713	0.471	0.198	1.74691E-95
TREM1	4.5796E-115	0.651591923	0.536	0.22	1.1791E-110
LGALS1	2.9119E-118	0.648098219	0.997	0.951	7.4972E-114
IGFBP7	2.3038E-157	0.643765345	0.421	0.121	5.9316E-153
CITED2	1.06403E-58	0.637133751	0.572	0.351	2.73955E-54
BSG	6.9088E-103	0.635216148	0.83	0.623	1.7788E-98
SBDS	5.8028E-107	0.625189537	0.625	0.332	1.4941E-102
RANBP1	1.08053E-99	0.623380736	0.766	0.497	2.78204E-95

LINC01272	2.6008E-123	0.617862577	0.703	0.339	6.6963E-119
WBP5	1.80007E-97	0.615975543	0.625	0.324	4.63465E-93
LGALS3	1.48769E-63	0.614416331	0.92	0.763	3.83036E-59
MMP19	3.42323E-75	0.613349905	0.481	0.234	8.81379E-71
PHLDA2	3.0944E-106	0.609340437	0.533	0.227	7.9672E-102
SLAMF9	1.7361E-136	0.6082891	0.359	0.097	4.4699E-132
IL4I1	1.17201E-55	0.606623547	0.561	0.336	3.01759E-51
RALA	1.75416E-95	0.606017494	0.623	0.34	4.51644E-91
VIM	6.6536E-119	0.605473312	0.992	0.911	1.7131E-114
HILPDA	7.23895E-79	0.603948753	0.244	0.074	1.86381E-74
CTSD	2.6367E-103	0.603276737	0.996	0.899	6.78862E-99
APOE	6.03315E-52	0.600792064	0.995	0.884	1.55336E-47
IL1RN	1.05764E-48	0.596622143	0.599	0.38	2.72311E-44
ADAM8	1.0565E-120	0.59626425	0.425	0.144	2.7202E-116
LMNA	4.38701E-67	0.594645574	0.74	0.493	1.12952E-62
VCX2	0	0.594029485	0.316	0.015	0
ALDOA	2.1993E-122	0.593986547	0.985	0.89	5.6625E-118
MT1M	1.41637E-14	0.587378966	0.139	0.073	3.64673E-10
CD59	1.91267E-73	0.582367033	0.774	0.524	4.92456E-69
APLP2	6.19249E-68	0.580641288	0.842	0.645	1.59438E-63
GPC3	1.0105E-170	0.575359178	0.378	0.093	2.6017E-166
S100A6	1.01958E-92	0.566518595	0.981	0.799	2.62511E-88
PLTP	6.98912E-99	0.564458156	0.724	0.404	1.79949E-94
DUSP2	6.79051E-34	0.562333533	0.673	0.496	1.74835E-29
ANG	4.3406E-123	0.560448526	0.536	0.216	1.1176E-118
EGR1	8.49869E-73	0.5597569	0.696	0.415	2.18816E-68
GEM	5.2355E-29	0.556328212	0.281	0.15	1.34798E-24
HSPA1A	7.74042E-91	0.556243508	0.973	0.793	1.99293E-86
PLAUR	2.05333E-47	0.556062333	0.893	0.719	5.28671E-43
ADAMDEC1	4.60038E-48	0.555148295	0.329	0.159	1.18446E-43
CYSTM1	2.80116E-66	0.555125297	0.674	0.454	7.21215E-62
CCL2	3.18306E-31	0.554613156	0.411	0.237	8.19542E-27
SH3BGRL3	1.39865E-73	0.551081656	0.987	0.954	3.6011E-69
S100A13	3.5809E-120	0.549610452	0.433	0.154	9.2198E-116
TPI1	6.3532E-114	0.544165906	0.983	0.892	1.6358E-109
PLA2G7	7.38974E-65	0.540761379	0.608	0.357	1.90264E-60
H2AFY	1.18575E-66	0.536139395	0.873	0.688	3.05294E-62
UBE2C	2.9067E-173	0.534741502	0.468	0.132	7.4838E-169
DAD1	2.2563E-78	0.53367129	0.848	0.651	5.8093E-74
VAPA	6.02846E-68	0.532044088	0.826	0.648	1.55215E-63

UBB	1.73353E-67	0.53128142	0.979	0.921	4.46333E-63
POLR2F	3.96962E-63	0.529855293	0.666	0.465	1.02206E-58
LAPTM4B	8.0745E-179	0.529622138	0.376	0.088	2.0789E-174
RPS27L	1.92963E-85	0.529552932	0.945	0.81	4.96822E-81
DNAJB1	2.78605E-75	0.523819676	0.915	0.713	7.17324E-71
RPS26	5.7009E-116	0.520599869	0.991	0.923	1.4678E-111
EMP3	1.43405E-74	0.520354053	0.919	0.722	3.69226E-70
MAP1LC3B	9.47884E-70	0.520079048	0.79	0.569	2.44052E-65
STOML2	2.81725E-61	0.519178022	0.575	0.366	7.25357E-57
WFDC2	0	0.51815175	0.289	0.018	0
DMKN	0	0.517968042	0.292	0.016	0
C15orf48	1.06728E-94	0.51760599	0.891	0.616	2.74792E-90
FBLN1	0	0.51536677	0.287	0.02	0
TMEM70	1.30536E-44	0.514259128	0.569	0.388	3.3609E-40
CAPG	3.78904E-63	0.512458291	0.902	0.708	9.75563E-59
EPCAM	0	0.506292552	0.293	0.018	0
NOP10	1.36059E-78	0.505155787	0.914	0.785	3.5031E-74
BEX1	0	0.504756054	0.269	0.014	0
CFD	6.80078E-46	0.502160259	0.665	0.436	1.751E-41
EIF6	1.07996E-65	0.500244488	0.702	0.489	2.78058E-61

Table S6: The The detailed quality information of eight ST samples.

sample	age	gender	stage	Number of Spots Under Tissue	Mean Reads per Spot	Mean Reads Under Tissue per Spot	Fraction of Spots Under Tissue	Reads Mapped to Probe Set	Reads Mapped Confidently to Probe Set	Fraction Reads in Spots Under Tissue	Median Genes per Spot	Median UMI Counts per Spot	Genes Detected
p1	49	male	I	4825	67236.18	66119.21	0.97	0.99	0.94	0.99	6218	19906	18056
p2	53	female	I	4258	75977.88	71074.36	0.85	0.99	0.99	0.94	6164	31282	18052
p3	51	female	II	4986	71070.03	70253.86	1	0.98	0.81	1	1566.5	19941	18040
p4	40	male	II	4692	74092.74	72944.5	0.94	0.99	0.98	0.99	7138.5	30512.5	18059
p5	53	female	I	4962	64385.89	63747	0.99	0.99	0.99	1	7729.5	38078.5	18047
p6	35	male	III	4990	69052.37	68482.48	1	0.99	0.98	1	5697.5	14317	18062
p7	56	female	III	4992	70420.55	69833.25	1	0.99	0.85	1	3544.5	8679.5	18072
p8	57	male	IV	4486	72496.57	71287.92	0.9	0.99	0.98	0.99	5199.5	11014	18050
